# Arsenic Resistance Protein BcArs2 Modulates Endogenous ROS Homeostasis in Infection Cushions to Enhance the Virulence of *Botrytis cinerea* Toward Host Plants

**DOI:** 10.1111/mpp.70359

**Published:** 2026-10-13

**Authors:** Yinshan Zhang, Huiqiang Feng, Xueting Chen, Guihua Li, Mingzhe Zhang, Qing‐Ming Qin

**Affiliations:** ^1^ College of Plant Science Jilin University Changchun China; ^2^ Department of Molecular Microbiology and Immunology, School of Medicine, Christopher S. Bond Life Sciences Center The University of Missouri Columbia Missouri USA

**Keywords:** *BcARS2*, *Botrytis cinerea*, catalase, H_2_O_2_, infection cushion, ROS, toxin

## Abstract

*Botrytis cinerea* triggers destructive grey mould with substantial economic losses, yet the functions of numerous pathogenicity genes remain largely unclear. Here, by screening a T‐DNA insertion library, we identified *BcARS2*, a key component of the Microprocessor complex. *BcARS2* is constitutively expressed, localizes to the nucleus, and modulates gene expression. The Δ*Bcars2* mutants exhibited impaired vegetative growth, enhanced conidiophore sporulation, increased conidiation, reduced sclerotial size, and increased sensitivity to multiple stresses. Pathogenicity assays showed that different inoculation methods led to distinct virulence phenotypes in Δ*Bcars2* mutants. Microscopic observations revealed that although the Δ*Bcars2* mutant formed normal appressoria and successfully invaded host cells, its invasive hyphae exhibited limited expansion. Furthermore, exogenous cAMP did not restore the impaired development of infection cushions (ICs), resulting in defective infection. Multitranscriptome analyses demonstrated that *BcARS2* positively regulates *BcPDI1* and *BcCGF1*, two key genes responsible for reactive oxygen species (ROS) homeostasis and highly expressed during the IC developmental stage. Correspondingly, deletion of *BcARS2* upregulated catalase gene expression, elevated catalase activity, reduced H_2_O_2_ accumulation, disturbed ROS balance, and ultimately inhibited IC formation. In addition, most toxin biosynthesis‐related genes, which are normally upregulated during IC development, were significantly downregulated in the Δ*Bcars2* mutant. Collectively, *BcARS2* modulates the virulence of *B. cinerea* by regulating IC development, vegetative hyphal growth, and stress responses.

## Graphical abstract

*BcARS2* negatively regulates catalase gene expression and CAT activity, and positively regulates *BcCGF1* to maintain intracellular H_2_O_2_ homeostasis. This mediates infection‑cushion development and facilitates toxin biosynthesis, ultimately governing the pathogenicity of *Botrytis cinerea.*

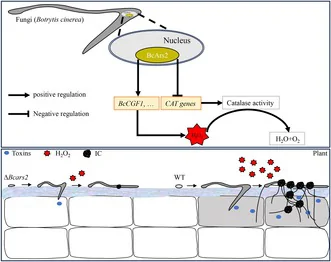

## 1 Introduction

*Botrytis cinerea*, ranked as the second most significant pathogenic fungus in plant pathology, has been extensively studied as a critical necrotrophic model organism (Dean et al. 2012). Notably, it infects more than 1000 plant species, including nearly all fruit and vegetable crops and many ornamental flowers, leading to global economic losses estimated at $10 to $100 billion annually (Fillinger and Elad 2016). Furthermore, *B. cinerea* can colonize diverse plant tissues including leaves, fruits, and stems, and inflicts severe damage both pre‐ and postharvest (Dean et al. 2012). *B. cinerea* possesses broad genetic variability and exceptional stress adaptability, enabling it to rapidly develop resistance to traditional fungicides (Sun et al. 2010; De Miccolis Angelini et al. 2014). Existing alternative control techniques all have inherent limitations. The large‐scale field efficacy and biosafety of animal‐ and plant‐derived active substances remain to be validated. Although plant‐derived compounds and antagonistic microorganisms exhibit promising application potential, their field control efficacy is unstable. Host resistance against *B. cinerea* is a quantitative trait, which poses substantial challenges for disease resistance breeding (Singh et al. 2024). To tackle this persistent challenge in agricultural production, in‐depth studies on the functions of pathogenicity genes in *B. cinerea* will provide key targets for developing novel eco‐friendly fungicides and theoretical support for breeding disease‐resistant crop varieties.

In agricultural fields, *B. cinerea* conidia land on plant surfaces via wind, rain, and agricultural operations. Under favourable conditions (moderate temperature, high humidity, and organic nutrient availability), they germinate and differentiate into specialized infectious structures including swollen‐tip appressoria and highly melanized infection cushions (ICs) to invade host tissues (Gourgues et al. 2004; Choquer et al. 2021). *B. cinerea* also secretes virulence factors, including cell wall‐degrading enzymes, toxins, effectors, and small RNAs (sRNAs), to exert its pathogenicity. For instance, the transcription factor BcXyr1, which regulates (hemi‐)cellulase production, is crucial for fungal pathogenicity (Ma et al. 2022). The fungus produces non‐host‐specific phytotoxins, including botcinic acid (BOA) and botrydial (BOT), to exert virulence (Porquier et al. 2019). Additionally, effector proteins such as BcXYG1 contribute to fungal virulence by inducing host cell death (Su et al. 2024). Furthermore, *B. cinerea* deploys sRNAs as effectors that translocate into host cells to silence plant defence genes via RNA interference (Weiberg et al. 2013; He et al. 2023). These pathogenesis‐related factors act synergistically to silence host immune response genes, kill host cells, and hijack host nutrients for their own growth and development.

Ars2 was initially named for its involvement in regulating organismal tolerance to sodium arsenite (Rossman and Wang 1999; Gruber et al. 2009). Gradually, researchers identified Ars2 as a component of the cleavage complex that participates in processing microRNAs. In plants (Michlewski and Cáceres 2019), miRNA‐coding genes are transcribed by RNA polymerase II in the nucleus to generate primary transcripts (pri‐miRNAs) of approximately 1 kb in length. The cap‐binding proteins CBP20/80 bind to the 5′ cap structure of pri‐miRNAs (Laubinger et al. 2008), while the RNA‐binding protein DAWDLE (DDL) associates with their basal regions, jointly promoting the formation of stable secondary structures in pri‐miRNAs (Yu et al. 2008). Subsequently, DICER‐LIKE 1 (DCL1) (Kurihara and Watanabe 2004), the zinc‐finger protein SERRATE (SE, also known as ArsS2) (Lobbes et al. 2006; Yang et al. 2006), the scaffold protein TOUGH (TGH) (Ren et al. 2012), and the double‐stranded RNA‐binding protein HYPONASTIC LEAVES 1 (HYL1) (Han et al. 2004; Wu et al. 2007) assemble into a cleavage complex. The cap‐binding proteins carrying pri‐miRNAs recruit this cleavage complex through interaction with Ars2 (Andersen et al. 2013). The complex first processes pri‐miRNAs into pre‐miRNAs, which are further cleaved to generate miRNA/miRNA* duplexes. The 3′‐terminal ribose of the miRNA/miRNA* duplex undergoes 2′‐O‐methylation catalysed by HUA ENHANCER 1 (HEN1) (Achkar et al. 2016). Subsequently, the miRNA guide strand is selectively loaded onto ARGONAUTE (AGO) proteins to assemble into the RNA‐induced silencing complex (RISC), which mediates target RNA cleavage and translational repression (Guo et al. 2014). Following these processing steps, bidirectional transport of small RNAs between pathogens and hosts mediates immune and virulence processes (Cai et al. 2018; He et al. 2023). Studies in *B. cinerea* have shown that overexpressing Bc‐siR37 in *Arabidopsis thaliana* leads to the suppression of target gene expression in the plant, thereby enhancing its sensitivity to *B. cinerea* (Wang et al. 2017). Weiberg et al. demonstrated that during infection, Bc‐siR3.2 can silence *Arabidopsis* targets *AtMPK1*, *AtMPK2*, and also tomato *SlMAPKKK4*, which are crucial proteins in plant immune signalling (Weiberg et al. 2013).

*ARS2* has been shown to play crucial roles in the pathogenesis of major diseases (Yuan et al. 2023). For instance, glioblastoma (GBM), a common malignant brain tumour, is highly aggressive and lethal. Research by Ke et al. revealed that *ARS2* was expressed at higher levels in four GBM cell lines than in a normal brain astrocyte cell line. Overexpression of *ARS2* promoted proliferation and colony formation in GBM cells, whereas *ARS2* knockdown inhibited these processes. Knockdown of *ARS2* suppressed the activation of the MAPK/ERK pathway, leading to G1‐phase cell cycle arrest in GBM cells and ultimately inhibiting cell proliferation (Ke et al. 2018). Chen et al. also reported that knockdown of *ARS2* reduced the expression of miR‐6798‐3p, thereby upregulating the levels of p53 and p21, inhibiting GBM growth, and prolonging the survival time of tumour‐bearing mice (Chen et al. 2018). Similar results were observed in acute myeloid leukaemia (AML) cell lines (Hu et al. 2019).

Research on Ars2 in plant‐pathogenic microorganisms is extremely limited. To date, relevant studies have been conducted only in *Fusarium graminearum*, where *FgARS2* has been shown to be involved in DNA damage repair, growth regulation, and virulence (Bui et al. 2019). However, exactly how *ARS2* regulates pathogenicity in phytopathogens remains unclear. In light of this, a thorough exploration of the functions of Ars2 in *B. cinerea* is not only highly innovative but also of great significance for the development of novel pesticides, as well as for the provision of cross‐species insights and references for studying the mechanisms underlying major diseases.

## 2 Results

### 2.1 Identification of the *BcARS2* Gene in *B. cinerea*

From an insertion mutant library, we identified a slow‐growing *B. cinerea* mutant T8752 that exhibited reduced virulence on tobacco, apricot, and green bean leaves (Figure 1A,B). TAIL‐PCR and sequencing of the T8752 strain revealed that the T‐DNA fragment was inserted in the 5′ untranslated region (UTR) of the gene *BCIN_02g01800* (Figure 1E). Reverse transcription‐quantitative PCR (RT‐qPCR) analysis showed that the transcription level of *BCIN_02g01800* was significantly downregulated in mutant T8752 compared with the wild‐type (WT) strain (Figure 1C). Phylogenetic analysis indicated that the protein encoded by *BCIN_02g01800* clustered into one clade with the protein encoded by *SS1G_01819* from *Sclerotinia sclerotiorum*, suggesting their close phylogenetic relationship (Figure S1A). Functional domain analysis further confirmed that *BCIN_02g01800* orthologs harbour a DUF3546 domain (uncharacterized functional domain) and an ARS2 domain (arsenite‐resistance protein domain) (Figure S1B). Given that *FGSG_01106* in *F. graminearum* has been designated *FgARS2*, we named *BCIN_02g01800* as *BcARS2* based on these bioinformatic analyses. The three‐dimensional (3D) structures of BcArs2 and FgArs2 predicted by SWISS‐MODEL display high similarity (Figure S1C).

**FIGURE 1:**
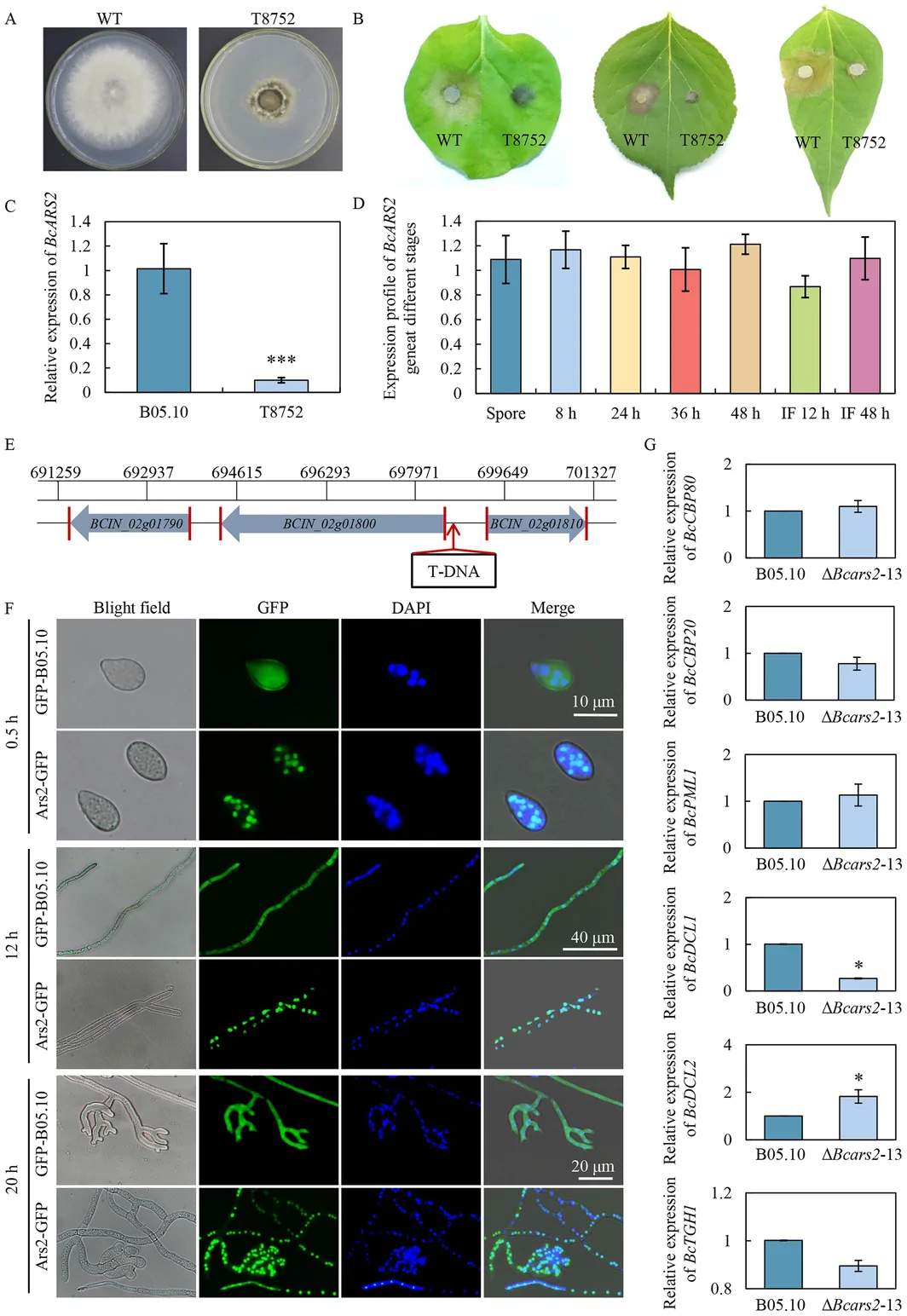
Identification of BcArs2 as a component of the cleavage complex. (A) Mycelial growth of *Botrytis cinerea* T8752 on potato dextrose agar in comparison to the wild type (WT). (B) Pathogenicity assays of T8752 on tobacco, apricot, and green bean leaves. (C) Expression level analysis of *BcARS2* (*BCIN_02g01800*) in WT (B05.10 and T8752). (D) Expression profile of *BcARS2* at different developmental stages. Vegetative growth stage: conidia (spore), conidial germination for 8, 24, 36, and 48 h; Infection stage (IF): Infection of green bean leaves for 12 and 48 h. (E) Schematic diagram showing the T‐DNA insertion position in the genome. (F) Nuclear localization of the BcArs2 protein in conidia (0.5 h), mycelia (12 h), and infection cushions (ICs, 20 h). GFP‐B05.10: Unfused GFP was transformed into the wild‐type (WT) strain. Ars2‐GFP strains: The Ars2::GFP fusion protein was introduced into the WT strain. Scale bar = 10 μm (conidia); 40 μm (mycelia); 20 μm (ICs). (G) Reverse transcription‐quantitative PCR validation of the expression of miRNA processing‐related genes in WT and Δ*Bcars2‐*13 strains. Data represent means ± standard deviations (SD) from at least three independent experiments, with triplicate samples examined for each treatment. Asterisks indicate significant differences based on Student's *t*‐test, *p < 0.05, ***p < 0.001.

Gene expression pattern and subcellular localization of proteins are closely related to their biological functions. To investigate the subcellular localization of BcArs2 in *B. cinerea* , we constructed a strain expressing BcArs2::GFP fusion protein, designated BcArs2‐GFP (Figure S2A). The control strain GFP‐B05.10 expressing unfused GFP was also generated (Figure S2B). Conidia, mycelia, and ICs of the Ars2‐GFP and GFP‐B05.10 strains were collected for fluorescence microscopy. The GFP signal in the Ars2‐GFP strain completely overlapped with the DAPI‐stained nuclei, whereas the unfused GFP signal in the control strain GFP‐B05.10 was distributed throughout the entire cell (Figure 1F). To analyse the expression pattern of *BcARS2* in *B. cinerea* , we collected samples from the vegetative growth stage (conidia and mycelia germinated for 8, 24, 36, and 48 h) and the infection stage (infected green bean leaves at 12 and 48 h post‐inoculation). RT‐qPCR analysis revealed that the expression level of *BcARS2* exhibited no significant differences across all time points examined (Figure 1D).

To investigate the biological functions of *BcARS2* in *B. cinerea* , we generated the Δ*Bcars2* knockout mutant and its complemented strain Δ*Bcars2*‐C using the strategy illustrated in Figure S2C,D. PCR was performed to detect the *BcARS2* gene in strains obtained during two rounds of single‐spore isolation, and no target band was amplified in all mutants (Figure S2E). Subsequently, Southern blot analysis using an *HPH* probe confirmed that the hygromycin resistance gene had been successfully integrated into the purified Δ*Bcars2*‐13 and Δ*Bcars2*‐27 strains, revealing the expected 3611 bp band. Subsequent hybridization with a *BcARS2*‐specific probe verified deletion of the target gene in both mutants, in contrast to the 1662 bp band observed in the WT strain (Figure S2F). Genomic PCR verification demonstrated that the *BcARS2* gene was completely deleted in the Δ*Bcars2* mutant but successfully restored in the Δ*Bcars2*‐C strain (Figure S2G). Semi‐quantitative PCR analysis further validated the absence of *BcARS2* expression in the Δ*Bcars2* strains and successful gene restoration in the Δ*Bcars2*‐C strain (Figure S2H).

During the processing of pri‐miRNAs into miRNA/miRNA* duplexes, CBC and DDL first stabilize the secondary structure of pri‐miRNAs. Subsequently, the interaction between CBC and ARS2 recruits the cleavage complex composed of ARS2, HYL1, DCL, and TGH proteins. Through bioinformatics analysis, we identified seven homologous components involved in miRNA processing in *B. cinerea* : cap‐binding protein complex (BcCbp20, BcCbp80), an RNA‐binding protein (BcDdl, also named BcPml1), and cleavage complex components including the arsenic resistance protein (BcArs2), ribonucleases (BcDcl1, BcDcl2), and the scaffold protein (BcTgh1). No homologue of HYL1 was identified. Deletion of *BcARS2* significantly downregulated the expression of *BcDCL1* and upregulated that of *BcDCL2*, whereas the transcript levels of *BcCBP20*, *BcCBP80*, *BcTGH1*, and *BcPML1* showed no significant differences (Figure 1G). Subsequent transcriptome sequencing further confirmed these expression patterns (Figure S3).

Overall, BcArs2, a stably expressed, nuclear‐localized component of the cleavage complex, regulates the expression of other complex components.

### 2.2 *BcARS2* Regulates Vegetative Hyphal Growth and Multifaceted Stress Responses

To investigate the role of *BcARS2* in vegetative growth, colony growth assays were performed with WT, Δ*Bcars2*, and Δ*Bcars2*‐C strains on complete medium (CM), using both conidial suspensions and mycelial plugs. Quantitative analysis revealed that the radial growth rate of Δ*Bcars2* mutants was reduced by approximately two‐thirds relative to the WT strain following incubation at 20°C (Figure 2A,B,E,F). This growth defect was fully restored in the Δ*Bcars2*‐C strain, indicating that *BcARS2* is essential for hyphal growth.

**FIGURE 2:**
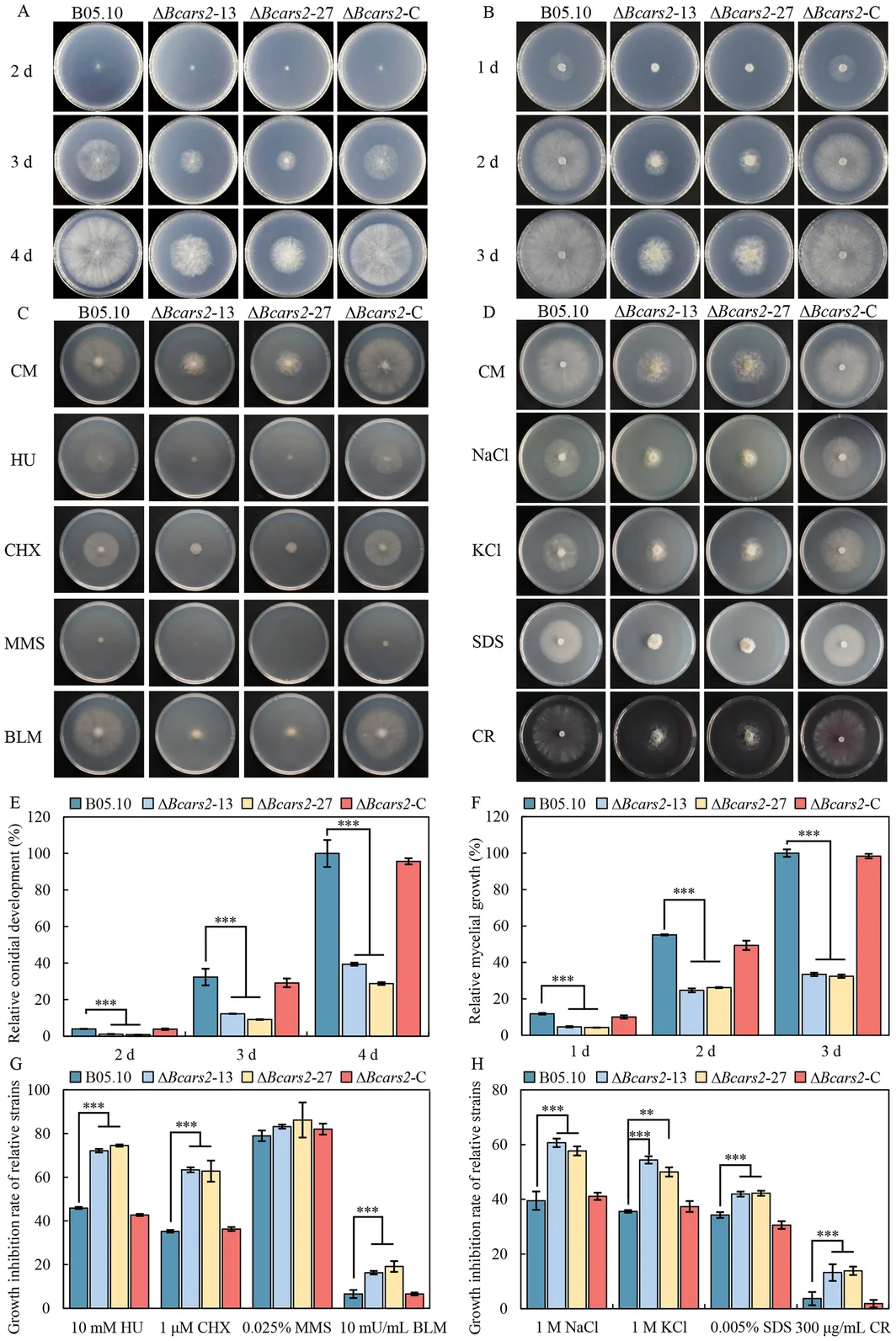
Growth and stress tolerance of the indicated strains. (A, B) Conidial development (A) and radial mycelial growth (B) of the indicated strains on complete medium (CM) plates incubated at 20°C in the dark. (C) Mycelial growth of the indicated strains on CM supplemented with DNA‐damaging compounds (10 mM hydroxyurea [HU], 0.025% methyl methanesulfonate [MMS], 10 mU/mL bleomycin [BLM]) and the translation inhibitor cycloheximide (CHX, 1 μM). (D) Mycelial growth of the indicated strains on CM supplemented with osmotic stress agents (1 M NaCl, 1 M KCl) or cell wall‐perturbing reagents (0.005% sodium dodecyl sulphate [SDS], 300 μg/mL Congo Red [CR]). (E, F) Quantitative analysis of conidial development (E) and radial mycelial growth (F) of the indicated strains cultured on CM plates. (G) Statistical analysis of mycelial growth inhibition of the indicated strains under treatment with DNA‐damaging compounds and the translation inhibitor. (H) Statistical analysis of mycelial growth inhibition of the indicated strains under treatment with abiotic stress factors. Data are presented as means ± SD from three independent experiments, with three plates analysed per strain in each experiment. Asterisks indicate significant differences based on Student's *t*‐test, **p < 0.01, ***p < 0.001.

As the carrier of genetic information, DNA is prone to damage induced by both endogenous and exogenous factors during replication, thereby leading to physiological defects in pathogenic fungi, such as cell cycle arrest and impaired growth and development. Given that *FgARS2* is critical for the DNA damage response in *F. graminearum* (Son et al. 2016; Bui et al. 2019), we next assessed whether *BcARS2* is involved in the DNA damage response in *B. cinerea* . Strains were exposed to three agents that induce distinct types of DNA damage (10 mM hydroxyurea [HU], 10 mU/mL bleomycin [BLM], 0.025% methyl methanesulponate [MMS]) and 1 μM of the translation inhibitor cycloheximide (CHX). HU induces replication stress, BLM elicits DNA double‐strand breaks, and MMS gives rise to base alkylation. The Δ*Bcars2* mutants exhibited significantly increased sensitivity to HU, BLM, and CHX relative to the control strains under identical conditions, whereas their sensitivity to MMS showed no obvious alteration (Figure 2C,G). These results suggest that deletion of *BcARS2*, analogous to *FgARS2* deletion, confers heightened sensitivity to specific DNA‐damaging and translational‐inhibition agents.

Pathogens encounter multifaceted environmental stresses during host invasion. To investigate the role of *BcARS2* in stress response in *B. cinerea* , the tested strains were inoculated on CM plates supplemented with distinct pathogenesis‐associated stress‐mimicking agents, including osmotic stress agents (NaCl/KCl) and cell wall‐damaging agents (SDS/Congo Red [CR]). The mutants exhibited significantly greater sensitivity than the WT and Δ*Bcars2*‐C strains to all stress conditions tested (Figure 2D,H), indicating that *BcARS2* participates in stress response pathways.

Collectively, deletion of *BcARS2* significantly repressed hyphal vegetative growth and markedly increased the sensitivity of the strain to osmotic stress, cell wall stress, DNA damage stress, and translational stress induced by CHX.

### 2.3 *BcARS2* Is Required for Reproductive Growth

Reproductive growth is essential for the survival, dispersal, and adaptation of microorganisms. To investigate *BcARS2* in this process, the test strains were inoculated on CM plates using spore suspensions or mycelial plugs. The Δ*Bcars2* mutants visibly initiated sporulation after 5 days of incubation, whereas WT and Δ*Bcars2*‐C strains did not produce any spores at this time. All strains eventually sporulated when the cultivation period was extended to 12 days (Figure 3A,B). Spore quantification revealed that the Δ*Bcars2* mutants produced significantly more spores than the WT and Δ*Bcars2*‐C strains at the corresponding time points (Figure 3E). Furthermore, the majority of Δ*Bcars2* spores exhibited abnormally shaped spores (Figure 3D,F). Germination assays indicated no significant differences in spore germination rates among the tested strains (Figure S4A,B). However, the germ tubes of the Δ*Bcars2* mutants were notably shorter than those of the control strains at each corresponding time point (Figure S4A,C). Further observation of conidiophores showed that Δ*Bcars2* strains displayed more sporogenous heads, a morphological trait linked to increased spore yield (Figure 3C).

**FIGURE 3:**
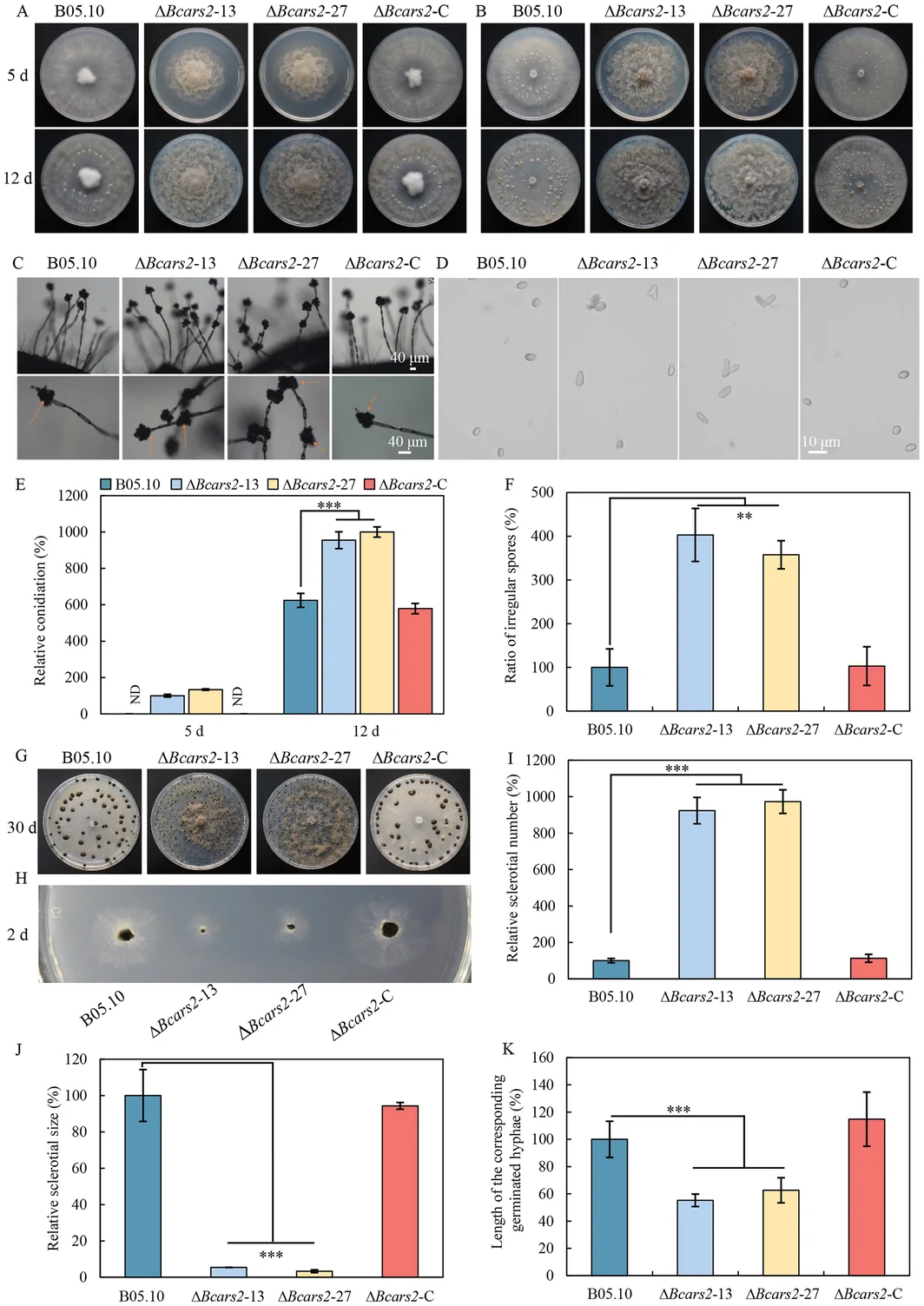
*BcARS2* is required for reproductive growth. (A, B) Complete medium (CM) plates were inoculated with conidia (A) or mycelial plugs (B). Conidial production of each strain was observed after 5 and 12 days. (C) Conidiophore morphology of the tested strains. Red arrows indicate conidiophore sporogenous heads. Scale bars = 40 μm. (D) Conidial morphology of the tested strains. Scale bar = 10 μm. (E) Quantification of relative conidiation of the tested strains on CM plates at the corresponding time points. (F) Proportion of abnormal conidia among the total conidia counted. (G) Sclerotial formation of the tested strains after incubation on CM plates for 30 days. (H) Sclerotial germination of the tested strains after 2 days. (I–K) Quantification of relative sclerotial number (I), size (J), and germinated hyphal length (K) of the tested strains. Data are presented as means ± SD from three independent experiments, with three plates analysed per strain per experiment. Asterisks indicate significant differences based on Student's *t*‐test, **p < 0.01, ***p < 0.001.

Sclerotia enable *B. cinerea* to survive harsh conditions and facilitate sexual reproduction. To assess *BcARS2*'s role in sclerotia formation, the WT, Δ*Bcars2*‐13, Δ*Bcars2*‐27, and Δ*Bcars2*‐C strains were cultured on CM plates at 20°C in darkness for 30 days. The Δ*Bcars2* mutants produced sclerotia that were only 10% of the WT in diameter but were 10‐fold more numerous. The Δ*Bcars2*‐C strain restored both sclerotial size and yield to WT levels (Figure 3G,I,J). When sclerotia were inoculated on CM, the miniature sclerotia of the Δ*Bcars2* mutants germinated normally (Figure 3H), whereas their emergent hyphae reached only half the length of those from the control strains (Figure 3K).

In summary, these results demonstrate that *BcARS2* has a pleiotropic function in regulating reproductive growth.

### 2.4 BcArs2 Regulates the Development of Infection Structures of *B. cinerea*

The appressoria and ICs are critical infection structures of *B. cinerea* that directly determine fungal penetration into host plant cells. To elucidate the role of BcArs2 in the formation of these structures, we compared their developmental dynamics across different strains using microscopic analysis. When conidial suspensions of the WT, Δ*Bcars2* mutants, and Δ*Bcars2*‐C strains were inoculated onto glass slides, the results revealed no statistically significant differences in appressorial formation among the strains (Figure 4A,B).

**FIGURE 4:**
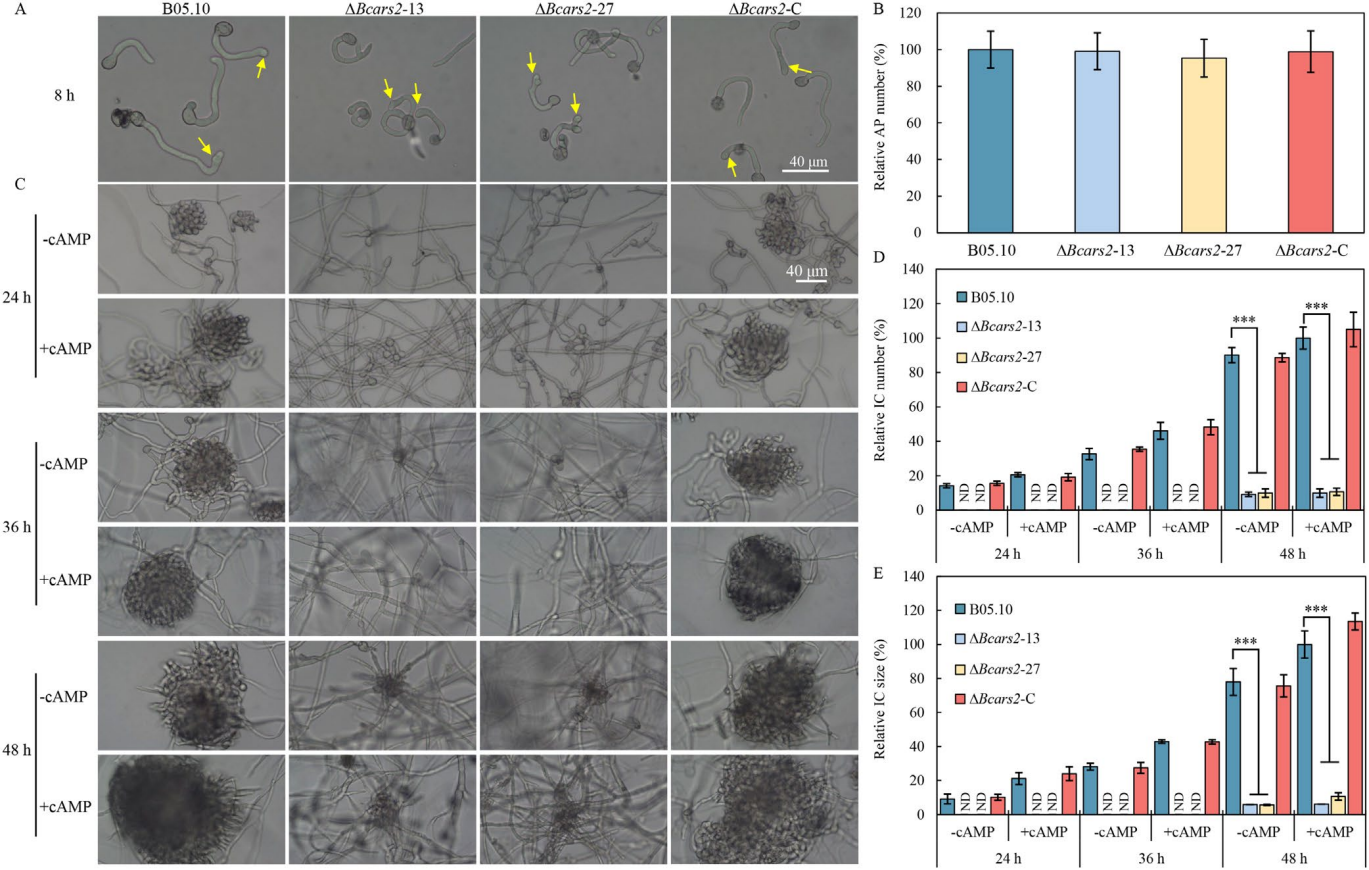
Development of infection structures in the indicated strains. (A) Appressorium formation in the indicated strains after 8 h. Yellow arrows indicate appressoria (AP). Scale bar = 40 μm. (B) Quantification of the relative number of appressoria (AP) in the indicated strains. (C) Development of infection cushions (ICs) in strains B05.10, Δ*Bcars2‐*13, Δ*Bcars2‐*27, and Δ*Bcars2*‐C at the indicated time points after inoculation. Scale bar = 40 μm. cAMP: cyclic adenosine monophosphate. (D) Quantification of the relative number of ICs in the indicated strains. (E) Quantification of the relative size of ICs in the indicated strains. Data represent means ± SD from three independent experiments, in which triplicate slides were examined for each strain per experiment. Asterisks indicate significant differences based on Student's *t*‐test, ***p < 0.001. ND indicates not detected.

We further incubated the samples under identical conditions for 24, 36, and 48 h to track the developmental progression of ICs. The Δ*Bcars2* mutants exhibited a complete loss of IC formation during the early (24 h) and mid‐stage (36 h) phases, with only a few immature structures detectable by the late stage (48 h) (Figure 4C). Quantitative analyses demonstrated that the mutants produced significantly fewer ICs, accounting for only approximately 10% of those produced by the WT and Δ*Bcars2*‐C strains, accompanied by a marked reduction in average size after 48 h (Figure 4D,E).

Given the well‐established regulatory role of the cAMP signalling pathway in fungal infection structure development (Sun et al. 2021), we examined whether exogenous cAMP could compensate for the developmental defects in the mutants. Supplementation of conidial suspensions with 50 μM cAMP significantly promoted IC maturation in both WT and Δ*Bcars2*‐C strains but failed to rescue IC formation and maturation in the Δ*Bcars2* mutants (Figure 4C–E).

Taken together, these results suggest that exogenous cAMP fails to restore the IC developmental defects in the Δ*Bcars2* mutant.

### 2.5 BcArs2 Is Required for Full Virulence in *B. cinerea*

Impaired development of ICs is frequently associated with reduced pathogenicity. Based on this association, we evaluated the pathogenicity of the WT, Δ*Bcars2*‐13, Δ*Bcars2*‐27, and Δ*Bcars2*‐C strains on two host plants using both conidial suspensions and mycelial plugs. Inoculation of detached green bean leaves with conidial suspensions revealed that the lesion expansion areas of the Δ*Bcars2* mutants at 36, 48, and 60 h post‐inoculation (hpi) were significantly smaller than those of the WT and Δ*Bcars2*‐C (Figure 5A,C). In mycelial plug inoculation assays, the mutants completely failed to form visible lesions over time (Figure 5B,D). This attenuated pathogenicity was also consistently observed on detached tobacco leaves (Figure S5A–D). Cellophane penetration assays were performed to mimic pathogen invasion into the host. Conidial suspensions were spread onto cellophane membrane. After incubation for 2 days, the cellophane was removed and incubation was continued for another 2 days, and all strains penetrated the cellophane (Figure 5F). In contrast, when inoculated with mycelial plugs under the same procedure, only the WT and Δ*Bcars2*‐C strains penetrated the cellophane, whereas the mutants failed to do so (Figure 5F). These results indicate that the Δ*Bcars2* mutant retains the ability to penetrate host tissues when inoculated as conidial suspensions, whereas its hyphae lose this penetration capacity.

**FIGURE 5:**
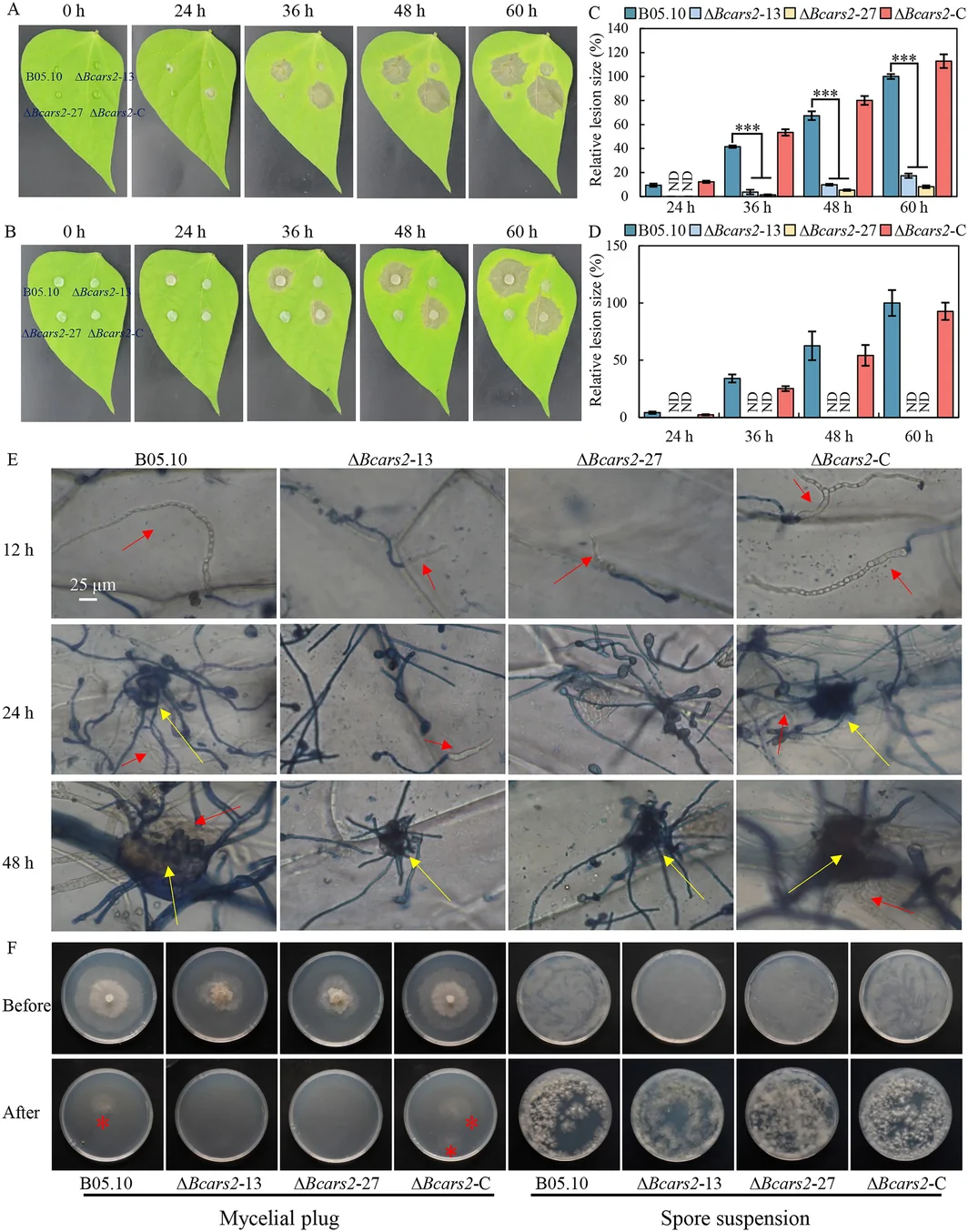
Pathogenicity assay of the indicated strains. (A, B) Disease symptoms on green bean leaves caused by the indicated strains during the infection time course via spore inoculation (A) or mycelial plug inoculation (B). (C, D) Quantification of lesion sizes caused by the indicated strains, as shown in (A) and (B). (E) Host penetration assay of onion epidermis inoculated with conidial suspensions of the indicated strains. Samples were stained with lactophenol blue and observed after 12, 24, and 48 h. Scale bar = 25 μm. Yellow arrows indicate infection cushions; red arrows indicate invasive hyphae. (F) Cellophane penetration assay using mycelial plugs or conidial suspension. “*” indicates penetrating hyphae. Data represent means ± SD from three independent experiments, in which triplicate leaves were examined for each strain per experiment. Asterisks indicate significant differences based on Student's *t*‐test, ***p < 0.001. ND indicates not detected.

To elucidate the pathogenic differentiation of the mutant caused by different inoculation methods, we inoculated onion epidermis with conidial suspensions and microscopically observed the infection process following lactophenol blue staining. The results showed that at 12 hpi, all strains successfully penetrated epidermal cells via appressoria; however, the invasive hyphae of the mutants were markedly shorter than those of the WT and Δ*Bcars2*‐C. By 24 h, the WT and Δ*Bcars2*‐C had developed ICs, whereas the mutants completely lacked such structures. Prolonged incubation until 48 h revealed that, in stark contrast to the mature ICs and robust invasive mycelia observed in the control strains, the mutants formed only immature ICs and failed to penetrate onion epidermal cells (Figure 5E).

When host plants are wounded, *B. cinerea* can directly invade through these wounds without relying on specialized infection structures, thereby minimizing the influence of such structures on disease progression. Inoculation assays using mycelial plugs on wounded green bean and tobacco leaves showed that the *BcARS2* deletion mutants could induce small lesions at wounded sites, whereas they failed to establish effective infection on non‐wounded tissues (Figure S6B,D,F,H). Similarly, when conidial suspensions were used for inoculation, the lesion areas on wounded tissues were significantly larger than those on non‐wounded tissues (Figure S6A,C,E,G).

Results from Sections 2.4 and 2.5 demonstrate that the mutant conidia can achieve limited host penetration via normally developed appressoria in the early stage. In contrast, when inoculated with mycelial plugs, the mutant relies on only a small number of immature ICs, which fail to effectively invade the host and consequently cannot produce lesions. Wounding of host tissues facilitated hyphal invasion and lesion formation by the mutant.

### 2.6 *BcARS2* Positively Regulates the Expression of Infection Cushion‐Associated Genes *BcPDI1* and *BcCGF1*

Given that IC development is a key factor limiting disease progression, and exogenous cAMP failed to restore IC maturity, we sought to elucidate how *BcARS2* mediates the regulation of IC formation. To this end, we performed transcriptome sequencing of the WT and Δ*Bcars2‐*13 strains at a late stage of IC development (48 hpi). The analysis identified 3628 significantly differentially expressed genes (DEGs) (|log_2_FoldChange| > 1, *p*_adj_ < 0.05), with 1478 upregulated and 2150 downregulated in the mutant (Figure S7A; Tables S2 and S3). KEGG enrichment analysis indicated that these DEGs were predominantly enriched in pathways related to secondary metabolite biosynthesis and eukaryotic ribosome biogenesis (Figure S7B). The DEGs identified from our WT/Δ*Bcars2*‐13 transcriptomic dataset were compared with the published DEGs from a microarray study of Choquer et al., which compared ICs with mycelia at 44 hpi (fold change ≤ −2 or ≥ 2; FDR < 0.05) (Figure 6A) (Choquer et al. 2021). A total of 631 overlapping genes were shared between the Δ*Bcars2*‐downregulated and IC‐upregulated profiles; conversely, 463 overlapping genes were identified between the Δ*Bcars2*‐upregulated and IC‐downregulated profiles (Figure 6B; Tables S4 and S5). Subsequent KEGG enrichment analysis revealed that the 631‐gene set was significantly enriched in metabolic and secondary metabolite biosynthesis pathways (Figure S7C; Table S4), whereas the 463‐gene set was enriched in the eukaryotic ribosome biogenesis pathway (Figure S7D; Table S5).

**FIGURE 6:**
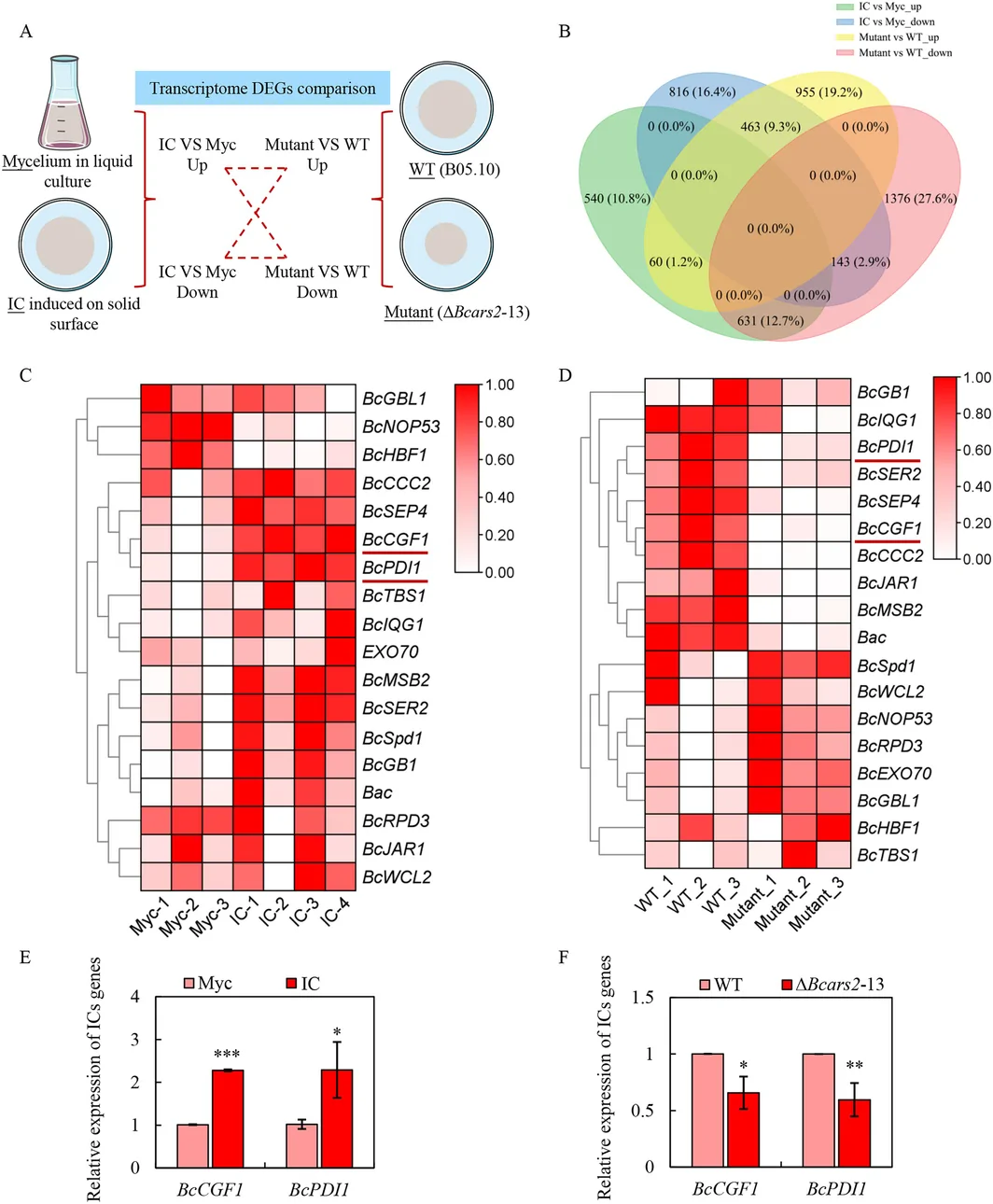
Multiple transcriptomic datasets revealed that *BcARS2* positively regulates *BcCGF1* and *BcPDI1*. (A) Schematic diagram of the analytical strategy for comparing differentially expressed genes (DEGs): DEGs between infection cushions (ICs) induced on solid surfaces and mycelia cultured in liquid medium, and DEGs between the wild‐type (WT) and Δ*Bcars2* strains in this study. (B) Venn diagram analysis comparing DEGs between ICs and mycelia with DEGs between the WT and Δ*Bcars2* strains in *Botrytis cinerea*. (C) Hierarchical clustering analysis of 18 previously reported *B. cinerea* IC development‐associated genes in ICs and mycelia. (D) Hierarchical clustering analysis of IC development‐associated genes in the WT and Δ*Bcars2* strains. (E, F) Validation of *BcCGF1* and *BcPDI1* expression levels via reverse transcription‐quantitative PCR in mycelia versus ICs (E) and in the WT versus Δ*Bcars2* strain (F). Data represent means ± SD from at least three independent experiments, with triplicate samples examined for each treatment. Asterisks indicate significant differences based on Student's *t*‐test, *p < 0.05, **p < 0.01, ***p < 0.001.

We screened 18 previously published *B. cinerea* genes associated with IC development, all of which exhibited phenotypic defects when knocked out, ranging from complete failure to form ICs to reductions in number and maturity. Heatmap analysis of the two transcriptomic datasets revealed that the expression levels of the protein disulfide isomerase gene *BcPDI1* and the conidial germination‐associated factor 1 gene *BcCGF1* were significantly higher at the IC formation stage than at the hyphal growth stage but markedly downregulated in the Δ*Bcars2* mutant (Figure 6C,D). This expression pattern was further confirmed by RT‐qPCR (Figure 6E,F). The expression levels, phenotypes, and relevant references of the remaining 16 genes in the two transcriptomes are presented in Table S6. Furthermore, *BcCGF1* and *BcPDI1* are associated with reactive oxygen species (ROS) homeostasis (Marschall and Tudzynski 2017; Zhang et al. 2020). In summary, integrated transcriptome analysis shows that *BcARS2* positively regulates the IC‐stage highly expressed *BcPDI1* and *BcCGF1*, with Δ*Bcars2* IC defects likely stemming from dysregulated ROS homeostasis.

### 2.7 BcArs2 Regulates IC Development by Modulating ROS Production

Does *BcARS2* mediate IC development by regulating ROS homeostasis? To test this hypothesis, we first performed a stress assay by supplementing CM with hydrogen peroxide (H_2_O_2_). The results demonstrated that the mycelial growth inhibition rate was significantly decreased in the Δ*Bcars2* mutants compared to the WT and Δ*Bcars2*‐C strains (Figure 7A,E). Microscopic observations revealed that compared with the control without H_2_O_2_ treatment, 7.5 mM H_2_O_2_ treatment markedly reduced the number and size of ICs in the WT and Δ*Bcars2*‐C strains (Figure S8A–C), whereas the mutant exhibited a significant increase in IC number (Figure S8A,B). These findings indicate that oxidative stress triggered by 7.5 mM exogenous H_2_O_2_ repressed mycelial growth and IC formation in the WT and Δ*Bcars2*‐C strains. Nevertheless, it compensated for endogenous ROS deficiency in the mutant and restored its capacity for IC development. The insensitivity of Δ*Bcars2* mutants to exogenous H_2_O_2_ prompted us to verify the correlation between *BcARS2*‐mediated IC formation and endogenous ROS production. Therefore, we employed 3,3′‐diaminobenzidine (DAB) staining to detect endogenous ROS levels in the WT, Δ*Bcars2‐*13, Δ*Bcars2‐*27, and Δ*Bcars2*‐C strains. In the presence of H_2_O_2_, DAB is oxidized to form an insoluble brown polymer. The reducing agent dithiothreitol (DTT) suppresses this reaction by scavenging H_2_O_2_, thereby preventing DAB oxidation. Mycelial plugs were inoculated onto CM lacking DTT and incubated for 48 h. Subsequent DAB staining showed that markedly less brown precipitate was observed in the Δ*Bcars2* mutants than in the WT and Δ*Bcars2*‐C strains. In contrast, the addition of DTT nearly abolished DAB staining in all strains (Figure 7B,F). To exclude the potential impact of differences in mycelial biomass, we quantified endogenous H_2_O_2_ using 0.1 g of mycelia. The results also confirmed a significant reduction in relative H_2_O_2_ production in the mutants (Figure 7C,G). Furthermore, we directly collected ICs for staining and microscopic observation. At 36 and 48 hpi, DAB staining of ICs showed a markedly reduced brown precipitate in Δ*Bcars2* mutants compared to controls. This reaction was abolished by DTT (Figure 7D,H). These findings verify that endogenous H_2_O_2_ accumulation is markedly reduced in ICs of Δ*Bcars2* mutants during their development.

**FIGURE 7:**
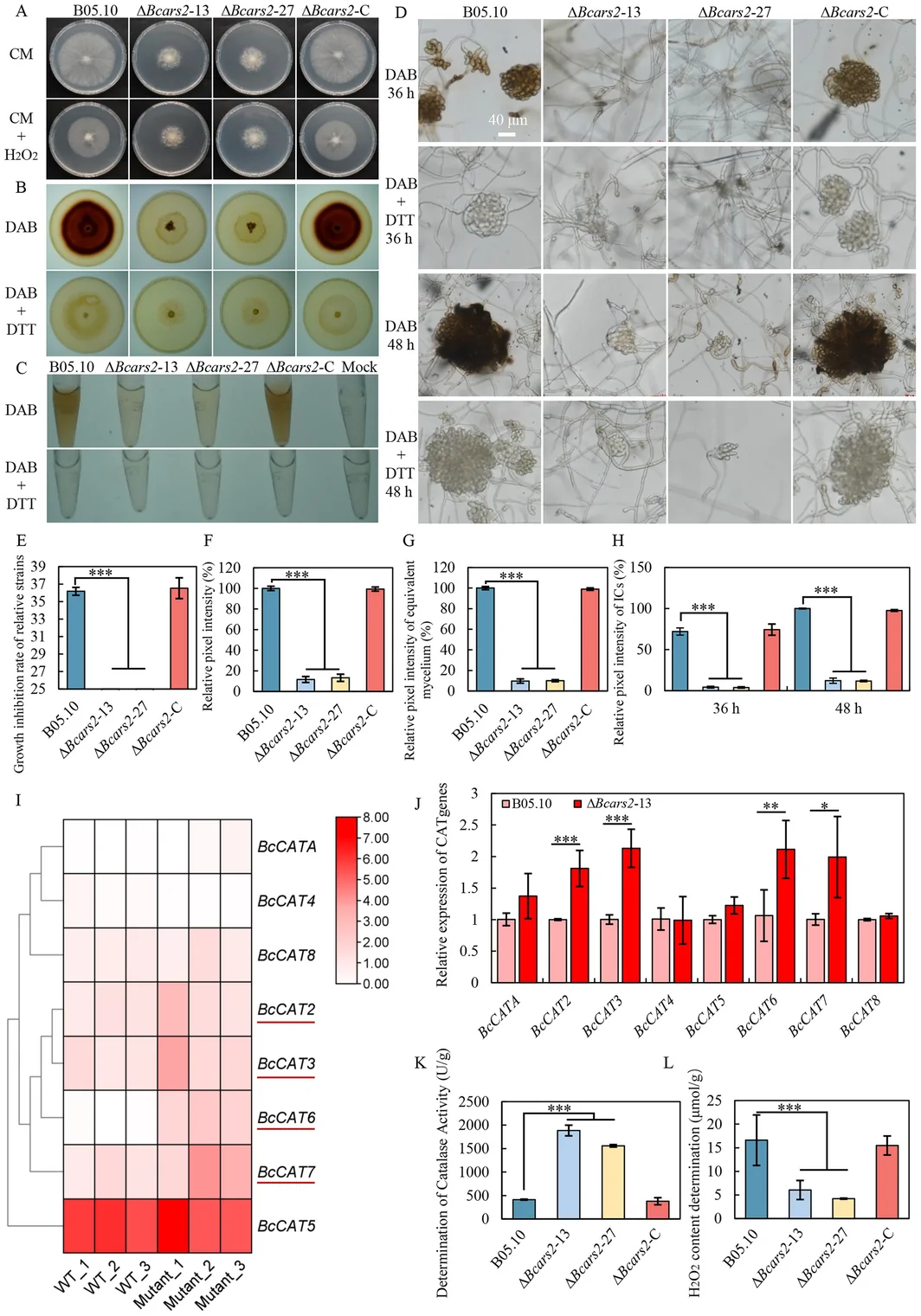
*BcARS2* regulates endogenous reactive oxygen species (ROS) production in infection cushions (ICs). (A) Mycelial growth of the indicated strains on complete medium (CM) plates supplemented with 7.5 mM H_2_O_2_. (B) Mycelial plugs of the wild‐type (WT, B05.10), Δ*Bcars2*, and Δ*Bcars2*‐C strains were inoculated onto CM plates without supplementation (upper panel) or supplemented with dithiothreitol (DTT) (lower panel), incubated at 20°C for 48 h, and subsequently stained with 3,3′‐diaminobenzidine (DAB) for 8 h. (C) DAB staining of 0.1 g mycelia from 48 h conidial germination cultures for 30 min (without DTT, upper panel; with DTT, lower panel). Mock: no mycelia added. (D) IC formation detected by DAB staining at 36 and 48 h post‐inoculation (hpi); DTT supplementation served as a control. Scale bar = 40 μm. (E) Statistical analysis of relative mycelial growth inhibition in the indicated strains, corresponding to (A). (F) Quantification of relative brown precipitate production based on mean pixel intensity in the tested strains, corresponding to (B, upper panel). (G) Quantification of relative brown precipitate production based on mean pixel intensity in the tested strains, corresponding to (C, upper panel). (H) Quantification of relative brown precipitate production based on mean pixel intensity in the tested strains, corresponding to (D). (I) Hierarchical clustering analysis of catalase‐encoding genes in the WT and Δ*Bcars2* strains. (J) Validation of catalase gene expression levels via reverse transcription‐quantitative PCR in the WT versus Δ*Bcars2* strains. (K) Determination of catalase activity in the tested strains. (L) Determination of H_2_O_2_ content in the tested strains. Data represent means ± SD from at least three independent experiments with three replicates per experiment. Asterisks indicate significant differences based on Student's *t*‐test, *p < 0.05, **p < 0.01, ***p < 0.001.

Appropriate ROS levels are essential for the normal development of *B. cinerea* . Catalase contributes to redox homeostasis by catalysing the decomposition of H_2_O_2_ into water and oxygen. Insufficient H_2_O_2_ availability impairs IC formation. Analysis of the expression levels of catalase‐encoding genes can indirectly reflect alterations in enzymatic activity. Bioinformatic analysis revealed that the catalase gene family in *B. cinerea* comprises eight members (*BcCATA*, *BcCAT2–BcCAT8*). Transcriptomic data and RT‐qPCR validation showed that four of these genes (*BcCAT2*, *BcCAT3*, *BcCAT6*, *BcCAT7*) were significantly upregulated in the Δ*Bcars2* mutant (Figure 7I,J), implying potential alterations in catalase activity in these strains.

To test this hypothesis, we collected mycelia from 48 h cultures for catalase activity assays and H_2_O_2_ content determination. The results demonstrated that catalase enzyme activity was significantly higher in the mutants than in the control strains (Figure 7K). Correspondingly, the H_2_O_2_ content in the mutant mycelia was significantly lower (Figure 7L).

In summary, deletion of *BcARS2* results in upregulation of catalase‐encoding genes, which elevates catalase activity and leads to excessive scavenging of intracellular H_2_O_2_. As a result, ROS levels are insufficient to support the proper formation and development of ICs. Thus, *BcARS2* functions to maintain ROS homeostasis during IC development by negatively regulating the transcription of catalase‐encoding genes.

### 2.8 IC Defects Critically Compromise Toxin Biosynthesis Gene Expression

Beyond its role in physical penetration, the IC of *B. cinerea* also serves as a “virulence factory” that synthesizes the critical phytotoxins botrydial (BOT) and botcinic acid (BOA), key virulence determinants of grey mould. Double mutants deficient in BOA and BOT production (*bcboa6*Δ *bcbot2*Δ) exhibit markedly reduced virulence (Dalmais et al. 2011). Bioinformatic analyses have defined the BOA biosynthetic gene cluster (16 genes, *BcBOA1–17*, excluding *BcBOA14*) and the BOT biosynthetic gene cluster (7 genes, *BcBOT1–7*) in this fungus (Porquier et al. 2016, 2019). This study aimed to elucidate the relationship between impaired IC development and the expression of these toxin gene clusters. To this end, we analysed multiple published transcriptomic datasets.

Zhu et al. (2023) demonstrated that sublethal treatment with 2% calcium propionate (CP) inhibits IC formation. Our re‐analysis of their transcriptome data (CP vs. control) revealed significant downregulation of *BcBOT1‐5*, *BcBOA1*, *BcBOA3*, *BcBOA4*, and *BcBOA13* (Figure 8A). Further work by Hou et al. (2020) showed that knockout of *BcJAR1* significantly suppressed IC formation. In the Δ*Bcjar1* mutant, the expression of *BcBOT1–7* was significantly downregulated at the early stage of IC formation 36 h compared to the WT (Figure 8B), and *BcBOT1, 2, 3, 4, 5*, and *7* remained markedly downregulated after 72 h (Figure 8C). These results indicate that IC defects are significantly associated with toxin gene expression. Whether deletion of the *BcARS2* gene similarly leads to downregulation of toxin biosynthesis‐related genes warrants further validation.

**FIGURE 8:**
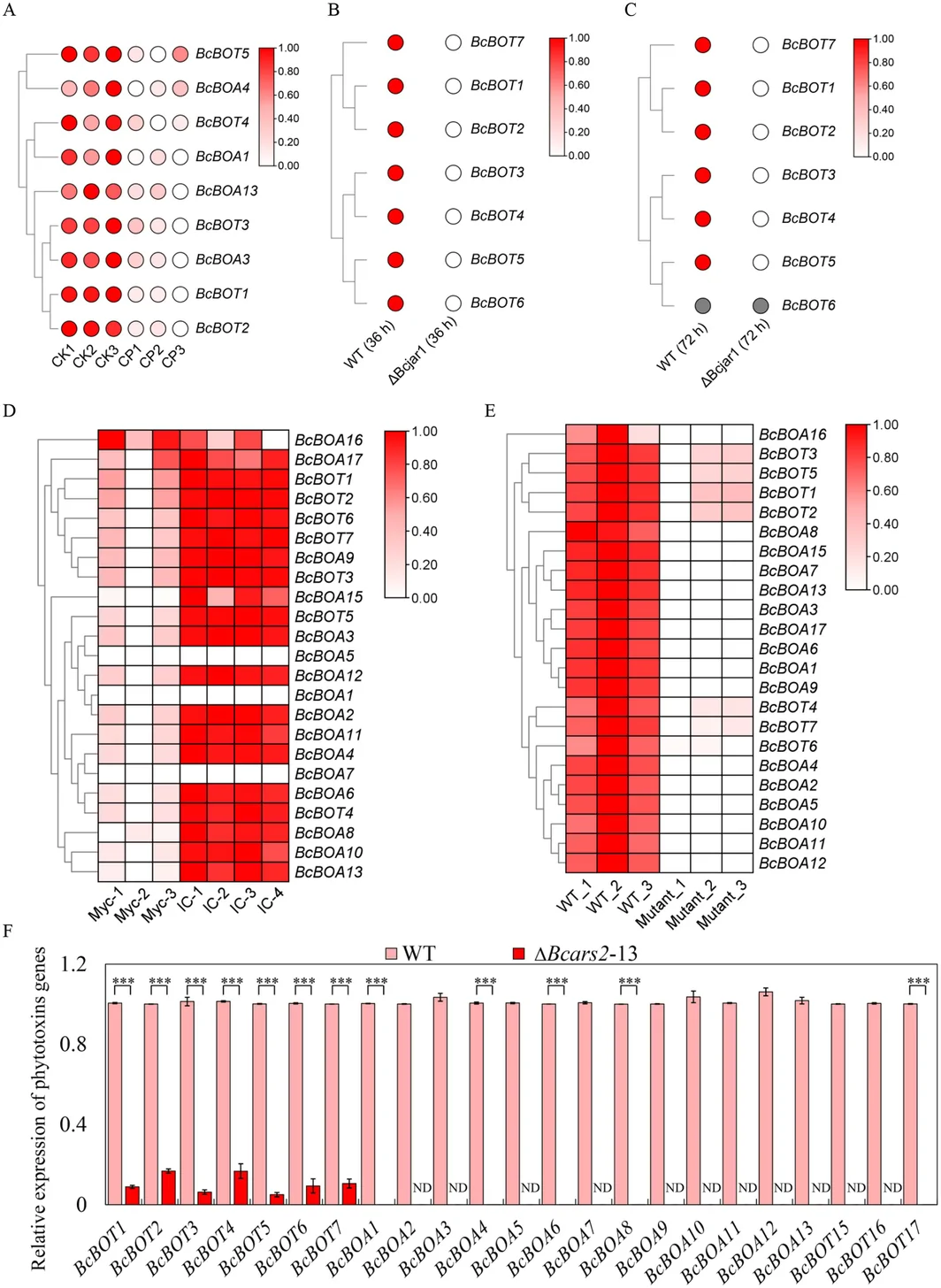
*BcARS2* positively regulates the expression of toxin biosynthesis‐related gene clusters. (A) Hierarchical clustering analysis reveals that botrydial (BOT) and botcinic acid (BOA) biosynthetic genes (*BcBOT1–5*, *BcBOA1*, *3*, *4*, *13*) are downregulated following treatment with 2% calcium propionate (CP). CK, negative control. (B) Hierarchical clustering analysis reveals that the Δ*Bcjar1* mutant downregulates the expression of *BcBOT1–7* genes at the early stage of infection cushion (IC) formation. WT, wild type. (C) Hierarchical clustering analysis reveals that the Δ*Bcjar1* mutant downregulates the expression of *BcBOT1–7* genes (excluding *BcBOT6*) at the late stage of IC formation. (D) Hierarchical clustering analysis shows upregulated expression of BOT and BOA biosynthetic gene clusters during IC formation in comparison to mycelial stage. Notably, *BcBOA1*, *5*, and *7* exhibited undetectable expression, whereas the expression changes of *BcBOA16*/*17* were not statistically significant. (E) Hierarchical clustering analysis shows downregulated expression of all BOT and BOA biosynthetic gene clusters in the Δ*Bcars2* mutant. (F) Validation of BOT and BOA biosynthetic gene cluster expression levels via reverse transcription‐quantitative PCR in WT versus Δ*Bcars2* strains. Data represent means ± SD from at least three independent experiments, with triplicate samples examined for each treatment. Asterisks indicate significant differences based on Student's *t*‐test, ***p < 0.001. ND indicates not detected.

By leveraging the published dataset of Choquer et al., we conducted a comprehensive analysis of these fungal toxin gene clusters. Within the BOA cluster (*BcBOA1–17*), 11 genes (*BcBOA2*, *3*, *4*, *6*, *8*, *9*, *10*, *11*, *12*, *13*, and *15*) were significantly upregulated during IC formation, whereas *BcBOA1*, *5*, and *7* exhibited undetectable expression, and the expression of *BcBOA16* and *17* was not significant. Correspondingly, all seven BOT biosynthetic genes (*BcBOT1–7*) were coordinately upregulated in developing ICs (Figure 8D). The reliability of this transcriptome data was further validated via RT‐qPCR analysis in Choquer et al.'s study. Transcriptomic analysis of the WT and Δ*Bcars2* mutant revealed that all genes within both the *BcBOA* and *BcBOT* biosynthetic clusters were significantly downregulated in the mutant (Figure 8E). This global repression in gene expression was further validated by RT‐qPCR assays (Figure 8F).

Collectively, our findings demonstrate that toxin biosynthesis‐related genes are specifically upregulated during IC development. Conversely, IC impairment—whether caused by deletion of *BcARS2*, CP‐mediated chemical inhibition, or disruption of *BcJAR1*—consistently induces significant downregulation of these genes. These results indicate that the IC acts as a spatiotemporal regulatory hub for toxin biosynthesis in *B. cinerea* .

## 3 Discussion

In the ongoing arms race between pathogens and hosts, elucidating the pathogenic mechanisms of the “crop killer” *B. cinerea* will advance fundamental understanding and guide crop production safety. This study reveals that the nuclear‐localized BcArs2 protein regulates multiple processes, including growth, development, asexual reproduction, stress adaptation, and pathogenicity. BcArs2 likely modulates hyphal development by influencing the DNA damage response and ribosome biogenesis. Deletion of *BcARS2* disrupts intracellular ROS homeostasis, thereby impairing IC formation. This developmental defect further results in global downregulation of toxin biosynthetic gene clusters, which in turn may drastically reduce virulence. Collectively, BcArs2 serves as a key virulence regulator in *B. cinerea* via multilayered regulatory pathways, representing a potential target for grey mould management.

The integrity and stability of DNA are crucial for organisms. However, DNA is highly susceptible to damage from both endogenous and exogenous sources, including single‐strand breaks (SSBs), double‐strand breaks (DSBs), base damage, and DNA‐protein crosslinks (DPCs) (Zhou and Elledge 2000). Unrepaired or mis‐repaired DNA lesions can trigger cell cycle arrest, cell death, loss of genetic information, and genomic instability (Jackson and Bartek 2009). To counter these threats, cells have evolved the DNA damage response (DDR) system. This system is intricately linked to DNA replication, gene transcription, DNA repair, and cell cycle checkpoints. It maintains genomic integrity and stability by detecting damage and coordinating repair mechanisms, thereby ensuring cell survival and normal cellular functions (Rouse and Jackson 2002). Treatment with three DNA‐damaging agents having distinct mechanisms revealed that Δ*Bcars2* and Δ*Fgars2* deletion mutants exhibit heightened sensitivity to HU, which induces replication stress, and BLM, which induces DSBs. In contrast, these mutants showed no sensitivity to MMS, which causes base alkylation damage (Son et al. 2016; Bui et al. 2019). This specific sensitivity pattern indicates that Ars2 may play a critical role in responding to replication stress and DSBs repair but may not directly participate in or significantly impact the base excision repair (BER) pathway. The absence of *BcARS2* impairs the cell's ability to respond to specific types of DNA damage via the DDR pathway and likely disrupts normal cell cycle progression. Cross‐species studies also indicate that Ars2 regulates basal growth by modulating the cell cycle. For instance, in human disease model cells (e.g., acute myeloid leukaemia cells, glioblastoma cells), *ARS2* deletion or knockdown causes cell cycle arrest at the G1 phase and significantly impairs cell proliferation (Ke et al. 2018; Hu et al. 2019). In *Arabidopsis thaliana* , aberrant expression of its homologue *AtSE* (overexpression accelerates growth; co‐suppression causes arrest) markedly alters plant growth rates (Prigge and Wagner 2001). These findings collectively indicate that Ars2 influences processes such as hyphal growth and development by regulating DDR and cell cycle progression. Furthermore, transcriptome analysis revealed that *BcARS2* deletion significantly impacts the ribosome biogenesis pathway. Concurrently, the Δ*Bcars2* mutants exhibit heightened sensitivity to CHX, an inhibitor of eukaryotic translation elongation. CHX blocks protein synthesis by specifically binding to the exit site (E‐site) of the 60S ribosomal subunit in eukaryotes, thereby inhibiting translation elongation factor eEF2‐mediated mRNA‐ribosome translocation. This impact on ribosome function and protein synthesis suggests that Δ*Bcars2* may fail to synthesize key cell cycle regulators (e.g., cyclins, CDKs) and other essential proteins in a timely manner, thereby exacerbating cell cycle dysregulation. In summary, deletion of *BcARS2* likely contributes to cell cycle dysregulation by affecting pathways such as DDR and protein synthesis, ultimately resulting in developmental abnormalities, including impaired growth in *B. cinerea* .

Regarding pathogenicity, the virulence of the Δ*Bcars2* mutant was significantly decreased relative to the WT and Δ*Bcars2*‐C strains. In agreement with this finding, other components involved in the miRNA processing pathway have also been reported to substantially regulate fungal pathogenicity. The attenuated virulence of the ∆*Bccbp20* mutants is likely associated with their hypersensitivity to environmental stresses, whereas ∆*Bccbp80* mutants appear to impair the pathogenic process primarily through defective IC formation (Zhang et al. 2025). Furthermore, Wang et al. (2016) demonstrated that host‐induced gene silencing (HIGS) targeting *BcDCL1* and *BcDCL2* in *Arabidopsis* and tomato significantly reduced the pathogenicity of *B. cinerea* . Our study indicates that knockout of *BcARS2* likely leads to attenuated virulence through a combination of multiple mechanisms.

Infection structures of *B. cinerea* , such as appressoria and ICs, are critical for host invasion (Fillinger and Elad 2016; Choquer et al. 2021), and their impairment significantly attenuates pathogen virulence (Marschall and Tudzynski 2017; Hou et al. 2020; Zhang, Sun, et al. 2020). In this study, the Δ*Bcars2* mutant formed normal appressoria but showed severe defects in IC development, as evidenced by significant reductions in both IC number and size. Microscopic examination showed that all strains formed appressoria within 8 h. On onion epidermis at approximately 12 hpi, Δ*Bcars2* mutants successfully penetrated the host via normal appressoria, yet their infection hyphae were markedly shorter than those of the control strains. By 24 h, the WT had developed ICs, which reached full maturity by 48 h. In contrast, the mutants exhibited immature IC development and failed to penetrate or produce invasive hyphae. Macroscopic pathogenicity assays confirmed these results. When inoculated with spores, Δ*Bcars2* mutants germinated normally, formed appressoria at an early stage, and successfully penetrated both tobacco and bean leaves to form lesions. However, the final lesion area was significantly smaller than that caused by the WT and complemented strains. In contrast, when inoculated with mycelial plugs, the mutants failed to produce visible lesions due to defective IC formation during later stages. Notably, when leaves were wounded prior to inoculation (a treatment allowing the pathogen to bypass infection structures), Δ*Bcars2* mutants were able to cause small lesions even when inoculated with mycelial plugs. Collectively, these results demonstrate that BcArs2 is required for normal IC development.

Experiments have demonstrated that exogenous cAMP application can restore defects in IC development in mutant strains (Zhang, Sun, et al. 2020; Sun et al. 2021). However, cAMP treatment failed to restore the ability of the Δ*Bcars2* mutants to form ICs on hydrophobic surfaces. This result indicates that cAMP is not a downstream effector of BcArs2, ruling out the possibility that BcArs2 regulates IC formation via the cAMP signalling pathway. Further analysis of 18 genes known to be involved in IC development showed that the expression of *BcPDI1* and *BcCGF1* was significantly elevated during the IC formation stage, and both genes were markedly downregulated in the Δ*Bcars2* mutant. Studies by Zhang et al. and Proual et al. further demonstrated that knockout of either *BcPDI1* or *BcCGF1* significantly inhibited IC formation, with both genes functioning through the ROS signalling pathway: BcPdi1 interacts with BcNoxA to regulate ROS levels (Marschall and Tudzynski 2017), whereas DAB and nitroblue tetrazolium (NBT) staining showed markedly reduced ROS accumulation in the ICs of the Δ*Bccgf1* mutant (Zhang, Sun, et al. 2020). The Δ*Bcars2* mutant downregulates two genes and exhibits reduced sensitivity to exogenous H_2_O_2_, suggesting that its IC formation defect arises from disrupted ROS homeostasis. We found that about half of the catalase genes were upregulated in the mutants, resulting in increased catalase activity, lower H_2_O_2_ levels, and weaker DAB staining in ICs. This aligns with prior findings where *BcWRKY22* overexpression activated *BcCAT2* and reduced H_2_O_2_ in Chinese cabbage (Wang et al. 2023). Thus, the upregulation of *CAT* in Δ*Bcars2* enhances H_2_O_2_ decomposition. The net effect in Δ*Bcars2* ICs is a sharp drop in H_2_O_2_ content—a level too low to sustain normal IC development.

Furthermore, knockout of *BcARS2* affected the expression of all toxin genes, which may reduce the contents of the two toxins in the mutant and thereby further attenuate pathogenicity. Reanalysis of the Choquer transcriptomic dataset showed that most BOT and BOA biosynthesis genes were significantly upregulated during IC development compared to the hyphal stage. When IC formation is disrupted, Δ*Bcars2* mutants exhibited even more pronounced suppression of toxin biosynthesis genes than CP treatment and the Δ*BcJar1* mutant (Hou et al. 2020; Zhu et al. 2023). As previous studies have reported, the double‐knockout mutant (Δ*Bcboa6*/*Bcbot2*), which completely lacks both BOT and BOA toxins, exhibits severely compromised virulence (Dalmais et al. 2011). We propose that the impaired development of ICs in the Δ*Bcars2* mutants constitutes an upstream regulatory event, leading to the coordinated downregulation of the entire suite of toxin biosynthesis genes. This might result in a substantial reduction in the accumulation of BOT and BOA, analogous to the effect of knocking out *BcBOA6*/*BcBOT2*, thereby attenuating mutants' pathogenicity. This however, requires direct validation through metabolomic analysis, specifically via the quantitative measurement of BOT and BOA levels in the Δ*Bcars2* mutants.

Finally, the homologues of BcArs2 play an indispensable role across multiple species, being essential for growth and development and critical for pathogenicity.

## 4 Experimental Procedures

### 4.1 Identification of *BcARS2*

Our laboratory constructed a T‐DNA insertion mutant library of *B. cinerea* comprising approximately 50,000 transformants using *Agrobacterium tumefaciens* ‐mediated transformation (ATMT) (Liu et al. 2019). Pathogenicity screening on green bean, tobacco, and apricot leaves identified a mutant strain, T8752, that exhibited reduced virulence. The flanking sequences of the T‐DNA insertion in T8752 were isolated and analysed by thermal asymmetric interlaced PCR (TAIL‐PCR) as previously described (Sun et al. 2021; Tang et al. 2024).

### 4.2 Phylogenetic and Structural Analysis of Protein Sequences

The DNA and protein sequences of *BcARS2* (*BCIN_02g01800*) were retrieved from the National Center for Biotechnology Information (NCBI, https://www.ncbi.nlm.nih.gov) and the *B. cinerea* B05.10 genome database on Ensembl Fungi (https://fungi.ensembl.org). Phylogenetic trees were generated using MEGA 11.0 (https://www.megasoftware.net) with maximum‐likelihood algorithms. The three‐dimensional structure was computationally modelled via SWISS‐MODEL (https://swissmodel.expasy.org) using automated homology detection. Functional domain architecture was predicted through InterPro database (http://www.ebi.ac.uk/interpro/) and the HMMPfam database (http://pfam.xfam.org/).

### 4.3 Fungal Developmental Assays

For each strain, a 5‐mm mycelial plug from the colony edge or 1 μL of a spore suspension (1 × 10^6^ spores/mL) was inoculated onto potato dextrose agar (PDA) and CM plates, respectively, followed by incubation at 20°C in the dark. Radial mycelial growth was documented from images taken on days 2, 3, and 4.

A 5‐mm‐diameter mycelial plug from each strain was transferred to a glass slide; hyphal morphology was then examined at 24 h, and conidiophore development was assessed at 72 h under a microscope.

To assess sporulation and germination, strains were inoculated on CM plates with mycelial plugs or spore suspensions. Conidia produced after 5 or 14 days were collected by washing with sterile water, counted with a haemocytometer, and observed for morphology. For germination assays, fresh suspensions were standardized to 5 × 10^5^ conidia/mL, combined 1:1 (v/v) with liquid CM containing 200 mg/L streptomycin, and 10 μL droplets were placed on glass slides for incubation at 20°C in the dark. Germination rates and germ tube lengths were quantified at 2, 4, and 6 h.

Spore suspensions (5 × 10^5^ conidia/mL) mixed 1:1 with CM containing 200 mg/L streptomycin were inoculated on slides (10 μL) and incubated at 20°C in darkness. Appressoria formation in strains B05.10, Δ*Bcars2*, and Δ*Bcars2*‐C was quantified at 8 hpi via microscope. ICs were analysed at 24, 36, and 48 hpi, with 50 μM cyclic adenosine monophosphate (cAMP) tested for rescuing Δ*Bcars2* defects.

Sclerotia formed on CM plates after 30‐day incubation were documented, with size and quantity recorded. They were then transferred to fresh CM plates to evaluate germination rates and mycelial growth length.

### 4.4 Fungal Cultivation, Transformation and Confirmation

*Botrytis cinerea* B05.10 (used as the genetic modification recipient) and its derived strains were cultured on PDA or CM at 20°C for 3–14 days.

The transformed strains were generated using established fungal genetic protocols (Feng et al. 2017; Sun et al. 2021). Briefly, the upstream and downstream flanking regions of *BcARS2* were amplified using primer pairs Ars2‐Up‐F/Ars2‐Up‐R and Ars2‐Down‐F/Ars2‐Down‐R, respectively. These PCR products were sequentially cloned into the pXEH vector (Sun et al. 2021) via XhoI/BglII and XbaI/PstI restriction enzyme sites. The resulting knockout construct was electroporated into *A. tumefaciens* AGL‐1 and introduced into B05.10 via ATMT. Positive transformants were selected on PDA supplemented with 50 μg/mL hygromycin B.

Two rounds of single‐spore isolation were performed to purify the obtained gene‐knockout mutants. Mutant strains were inoculated onto hygromycin B‐supplemented PDA plates and cultured at 20°C in the dark until sporulation. Conidia were harvested with sterile water, serially diluted, and spread onto selective water‐agar plates. After incubation at 20°C for 12–16 h, four germinated single conidia were picked under a stereomicroscope and transferred to selective PDA plates to yield single colonies. The above procedure was repeated for the second round of single‐spore isolation. The primer pair Ars2‐NF/Ars2‐NR was used to detect the *BcARS2* gene in strains collected at each purification stage. Mutants purified after two rounds of single‐spore isolation were used for subsequent experimental validation.

Gene replacement was confirmed by Southern blot analysis of AgeI‐digested genomic DNA, which was hybridized with probes synthesized using a DIG‐High Prime DNA Labeling and Detection Kit (Roche) according to published methods (Zhang, Wang, et al. 2020).

To generate the complemented strain, a PCR fragment carrying the native promoter, the full length of the *BcARS2* coding sequence, and the terminator was amplified using primers C‐Ars2‐F/C‐Ars2‐R. This fragment was cloned into the pSULPH vector (Sun et al. 2021) using the SpeI and PstI restriction sites. The resulting construct was introduced into Δ*Bcars2* via ATMT. Positive transformants were selected on PDA medium containing 100 μg/mL chlorimuron ethyl and were analysed by RT‐qPCR to measure *BcARS2* expression levels with primers RT‐Ars2‐F/RT‐Ars2‐R, using the *BcACT* gene, amplified with primers BcACT‐F and BcACT‐R, as the reference gene. In addition, the test strains were verified at the genomic level. Genomic DNA was extracted from the mycelia of WT, Δ*Bcars2* knockout mutant, and Δ*Bcars2*‐C complemented strains. The primer pair Ars2‐NF/Ars2‐NR was used for the specific PCR amplification of the *BcARS2* gene. PCR products were separated and visualized via 1% agarose gel electrophoresis.

The *Oilc* promoter, the *BcARS2* coding sequence, and GFP fused to the *TrpC* terminator were individually amplified via PCR and ligated into the pSULPH vector to generate the BcArs2‐GFP fusion construct (Zhang et al. 2025). The construct was introduced into the WT strain via ATMT. As a control, a construct containing the *Oilc* promoter driving GFP with the *TrpC* terminator was also generated in the pSULPH vector and subsequently transformed into the WT strain. To determine the subcellular localization of BcArs2, samples of spores, hyphae, and ICs from the respective strains were examined by fluorescence microscopy. The GFP signal was captured through the GFP channel, and nuclei were counterstained with 4′,6‐diamidino‐2‐phenylindole (DAPI, Sangon Biotech). All primers used in this study are listed in Table S1.

### 4.5 Plant Infection Assays

Fresh conidial suspensions (1 × 10^6^ spores/mL) of B05.10, Δ*Bcars2*‐13, Δ*Bcars2*‐27, and Δ*Bcars2‐*C were mixed 1:1 (v/v) with liquid CM. Aliquots (15 μL) of the mixture were inoculated onto green bean and tobacco leaves. In parallel, 5‐mm mycelial plugs from test strains were inoculated onto detached green bean and tobacco leaves. Lesion areas were measured at the designated time points. For wound inoculation assays, detached leaves were gently wounded with sterile pipette tips and inoculated with mycelial plugs or conidial suspensions as described above. Additionally, droplets of conidial suspension were applied to onion inner epidermal cells and incubated at 20°C under high humidity. At the designated time points, the inoculated epidermal cells were stained with lactophenol blue for 5 min and then examined under a microscopic imaging system to assess host‐pathogen interaction sites and penetration efficiency.

### 4.6 Stress Adaptation Assays

Mycelial plugs or spore suspensions of the tested strains were inoculated onto CM agar plates amended with various stressors, including osmotic agents (0.6 M NaCl or KCl; Sangon Biotech), an oxidative stress agent (7.5 mM hydrogen peroxide [H_2_O_2_]; Aladdin), cell wall‐perturbing agents (0.005% SDS and 500 μg/mL CR; Sangon Biotech), the translation inhibitor CHX (1 μM; Sangon Biotech), and DNA‐damaging compounds (0.025% MMS, 10 mM HU, and 10 mU/mL BLM; Sangon Biotech). All plates were incubated at 20°C in darkness for 72 h. Mycelial growth was assessed by measuring colony diameters, which were photographed for documentation. The experiment was performed with three technical replicates per treatment and was independently repeated three times.

### 4.7 Sample Preparation for RNA‐Seq Analysis

Spore suspensions (1 × 10^6^ spores/mL, 200 μL) of the B05.10 and Δ*Bcars2*‐13 strain were inoculated onto cellophane‐overlaid CM plates and incubated at 20°C for 48 h to induce IC formation. Three independent biological replicates were established, each containing five technical parallel samples. All samples were processed by Novogene Co. Ltd. for RNA extraction and library construction (https://cn.novogene.com/). RNA integrity was assessed using the RNA Nano 6000 Assay Kit of the Bioanalyzer 2100 system (Agilent Technologies). Following library construction and quality control verification, qualified libraries were pooled proportionally based on their validated concentrations and required sequencing depth, followed by Illumina sequencing to generate 150 bp paired‐end reads. Differential expression analysis between groups was performed using the DESeq2 R package (1.20.0) (Anders and Huber 2010).

### 4.8 qRT‐qPCR

Samples of the B05.10 and Δ*Bcars2*‐13 strains were collected according to transcriptomic sequencing protocols. For the collection of mycelial and infection cushion samples, a conidial suspension (1 × 10^6^ spores/mL, 200 μL) of the WT strain was inoculated onto cellophane‐overlaid PDA plates and incubated statically at 20°C. Mycelia were collected after 12 h of incubation, and ICs were harvested after 48 h. Total RNA was reverse‐transcribed using the PrimeScript RT Reagent Kit (TaKaRa) with 1 μg input, and analysed via qPCR with SYBR Green I (TaKaRa). The expression data were normalized to the actin gene *BcACT1*. Gene expression was quantified using the 2^−ΔΔ*C*t^ method. All experimental details followed established protocols from prior publications (Zhang, Sun, et al. 2020; Zhang et al. 2024).

### 4.9 Detection of ROS Production

ROS production during infection structure development was quantified according to established protocols (Cao et al. 2018; Hou et al. 2020), with ImageJ software for signal quantification (http://rsbweb.nih.gov/ij/). The spore suspension of the tested strain was spread on CM covered with cellophane. After 48 h, the mycelia were harvested and adjusted to a weight of 0.1 g. H_2_O_2_ content and catalase activity were measured using commercial assay kits (Solarbio; BC3590 and BC0205, respectively) in accordance with the manufacturer's instructions.

### 4.10 Statistical Analysis

Heatmaps were generated using TBtools (Chen et al. 2023). Quantitative data were derived from three biological replicates, each consisting of at least three technical replicates. For comparative analysis, control data (e.g., mycelial growth and lesion size) were normalized to 1 (or 100%) within each independent experiment. Statistical significance (*p* < 0.05) was determined using Student's *t*‐test.

## Author Contributions

**Yinshan Zhang:** conceptualization, methodology, formal analysis, visualization, writing – original draft. **Huiqiang Feng:** conceptualization, methodology, formal analysis, visualization, investigation. **Xueting Chen:** investigation, methodology. **Guihua Li:** conceptualization, project administration, investigation, methodology. **Mingzhe Zhang:** conceptualization, methodology, formal analysis, writing – review and editing, funding acquisition. **Qing‐Ming Qin:** conceptualization, methodology, funding acquisition, writing – review and editing, formal analysis.

## Funding

This research was funded by the Education Department Foundation of Jilin Province (No. JJKH20231198KJ), the National Natural Science Foundation of China (Grant Nos. 32001856, 31871913).

## Conflicts of Interest

The authors declare no conflicts of interest.

## Supporting information


**Figure S1:** Bioinformatics analysis of Ars2.

**Figure S2:** Construction and identification of *BcARS2* knockout, complementation, and GFP fusion strains.

**Figure S3:** Cluster analysis of the expression of miRNA processing‐related genes upon *BcARS2* knockout.

**Figure S4:** Conidial germination of the indicated strains.

**Figure S5:** Pathogenicity assay on tobacco leaves.

**Figure S6:** Pathogenicity analysis of the indicated strains on wounded and unwounded leaf tissues.

**Figure S7:** Analysis of differentially expressed genes (DEGs).

**Figure S8:** Exogenous hydrogen peroxide promotes infection‐cushion maturation in the mutant.

**Table S1:** Primers used in this study.

**Table S2:** Mutant vs. WT_DEG_down.

**Table S3:** Mutant vs. WT_DEG_up.

**Table S4:** IC vs. Myc_up & Mutant vs. WT_down.

**Table S5:** IC vs. Myc_down & Mutant vs. WT_up.

**Table S6:** 18 genes associated with infection cushion development.

## Data Availability

The data that support the findings of this study are available from the corresponding author upon reasonable request.

## References

[mpp70359-bib-0001] Achkar, N. P. , D. A. Cambiagno , and P. A. Manavella . 2016. “miRNA Biogenesis: A Dynamic Pathway.” Trends in Plant Science 21: 1034–1044. 10.1016/j.tplants.2016.09.003. 27793495

[mpp70359-bib-0002] Anders, S. , and W. Huber . 2010. “Differential Expression Analysis for Sequence Count Data.” Genome Biology 11: 106. 10.1186/gb-2010-11-10-r106. PMC3218662 20979621

[mpp70359-bib-0003] Andersen, P. R. , M. Domanski , M. S. Kristiansen , et al. 2013. “The Human Cap‐Binding Complex Is Functionally Connected to the Nuclear RNA Exosome.” Nature Structural and Molecular Biology 20: 1367–1376. 10.1038/nsmb.2703. PMC3923317 24270879

[mpp70359-bib-0004] Bui, D. C. , J. E. Kim , J. Shin , et al. 2019. “ *ARS2* Plays Diverse Roles in DNA Damage Response, Fungal Development, and Pathogenesis in the Plant Pathogenic Fungus *Fusarium graminearum* .” Frontiers in Microbiology 10: 2326. 10.3389/fmicb.2019.02326. PMC6803386 31681199

[mpp70359-bib-0005] Cai, Q. , L. L. Qiao , M. Wang , et al. 2018. “Plants Send Small RNAs in Extracellular Vesicles to Fungal Pathogen to Silence Virulence Genes.” Science 360: 1126–1129. 10.1126/science.aar4142. PMC6442475 29773668

[mpp70359-bib-0006] Cao, S. N. , Y. Yuan , Y. H. Qin , et al. 2018. “The Pre‐rRNA Processing Factor Nop53 Regulates Fungal Development and Pathogenesis via Mediating Production of Reactive Oxygen Species.” Environmental Microbiology 20: 1531–1549. 10.1111/1462-2920.14082. 29488307

[mpp70359-bib-0007] Chen, C. J. , Y. Wu , J. W. Li , et al. 2023. “TBtools‐II: A ‘One for All, All for One’ Bioinformatics Platform for Biological Big‐Data Mining.” Molecular Plant 16: 1733–1742. 10.1016/j.molp.2023.09.010. 37740491

[mpp70359-bib-0008] Chen, Y. B. , X. Y. Hu , Y. N. Li , et al. 2018. “Ars2 Promotes Cell Proliferation and Tumorigenicity in Glioblastoma Through Regulating *miR‐6798‐3p* .” Scientific Reports 8: 15602. 10.1038/s41598-018-33905-x. PMC6197286 30349053

[mpp70359-bib-0009] Choquer, M. , C. Rascle , I. R. Gonçalves , et al. 2021. “The Infection Cushion of *Botrytis cinerea*: A Fungal ‘Weapon’ of Plant‐Biomass Destruction.” Environmental Microbiology 23: 2293–2314. 10.1111/1462-2920.15416. 33538395

[mpp70359-bib-0010] Dalmais, B. , J. Schumacher , J. Moraga , et al. 2011. “The *Botrytis cinerea* Phytotoxin Botcinic Acid Requires Two Polyketide Synthases for Production and Has a Redundant Role in Virulence With Botrydial.” Molecular Plant Pathology 12: 564–579. 10.1111/j.1364-3703.2010.00692.x. PMC6640383 21722295

[mpp70359-bib-0011] De Miccolis Angelini, R. M. , C. Rotolo , M. Masiello , D. Gerin , S. Pollastro , and F. Faretra . 2014. “Occurrence of Fungicide Resistance in Populations of *Botryotinia fuckeliana* (*Botrytis cinerea*) on Table Grape and Strawberry in Southern Italy.” Pest Management Science 70: 1785–1796. 10.1002/ps.3711. 24338954

[mpp70359-bib-0012] Dean, R. , J. A. L. Van Kan , Z. A. Pretorius , et al. 2012. “The Top 10 Fungal Pathogens in Molecular Plant Pathology.” Molecular Plant Pathology 13: 414–430. 10.1111/j.1364-3703.2012.00822.x. PMC6638784 22471698

[mpp70359-bib-0013] Feng, H. Q. , G. H. Li , S. W. Du , et al. 2017. “The Septin Protein Sep4 Facilitates Host Infection by Plant Fungal Pathogens via Mediating Initiation of Infection Structure Formation.” Environmental Microbiology 19: 1730–1749. 10.1111/1462-2920.13613. 27878927

[mpp70359-bib-0014] Fillinger, S. , and Y. Elad . 2016. Botrytis—The Fungus, the Pathogen and Its Management in Agricultural Systems. Springer International Publishing. 10.1007/978-3-319-23371-0.

[mpp70359-bib-0015] Gourgues, M. , A. Brunet‐Simon , M. H. Lebrun , and C. Levis . 2004. “The Tetraspanin BcPls1 Is Required for Appressorium‐Mediated Penetration of *Botrytis cinerea* Into Host Plant Leaves.” Molecular Microbiology 51: 619–629. 10.1046/j.1365-2958.2003.03866.x. 14731267

[mpp70359-bib-0016] Gruber, J. J. , D. S. Zatechka , L. R. Sabin , et al. 2009. “Ars2 Links the Nuclear Cap‐Binding Complex to RNA Interference and Cell Proliferation.” Cell 138: 328–339. 10.1016/j.cell.2009.04.046. PMC2717034 19632182

[mpp70359-bib-0017] Guo, W. , J. Y. Liew , and Y. A. Yuan . 2014. “Structural Insights Into the Arms Race Between Host and Virus Along RNA Silencing Pathways in *Arabidopsis thaliana* .” Biological Reviews of the Cambridge Philosophical Society 89: 337–355. 10.1111/brv.12057. 24034134

[mpp70359-bib-0018] Han, M. H. , S. Goud , L. Song , and N. Fedoroff . 2004. “The *Arabidopsis* Double‐Stranded RNA‐Binding Protein HYL1 Plays a Role in microRNA‐Mediated Gene Regulation.” Proceedings of the National Academy of Sciences of the United States of America 101: 1093–1098. 10.1073/pnas.0307969100. PMC327156 14722360

[mpp70359-bib-0019] He, B. Y. , H. Wang , G. S. Liu , et al. 2023. “Fungal Small RNAs Ride in Extracellular Vesicles to Enter Plant Cells Through Clathrin‐Mediated Endocytosis.” Nature Communications 14: 4383. 10.1038/s41467-023-40093-4. PMC10359353 37474601

[mpp70359-bib-0020] Hou, J. , H. Q. Feng , H. W. Chang , et al. 2020. “The H3K4 Demethylase Jar1 Orchestrates ROS Production and Expression of Pathogenesis‐Related Genes to Facilitate *Botrytis cinerea* Virulence.” New Phytologist 225: 930–947. 10.1111/nph.16200. 31529514

[mpp70359-bib-0021] Hu, X. Y. , S. N. Xu , Y. B. Chen , et al. 2019. “Depletion of Ars2 Inhibits Cell Proliferation and Leukemogenesis in Acute Myeloid Leukemia by Modulating the miR‐6734‐3p/p27 Axis.” Leukemia 33: 1090–1101. 10.1038/s41375-018-0301-z. PMC6756072 30518811

[mpp70359-bib-0022] Jackson, S. P. , and J. Bartek . 2009. “The DNA‐Damage Response in Human Biology and Disease.” Nature 461: 1071–1078. 10.1038/nature08467. PMC2906700 19847258

[mpp70359-bib-0023] Ke, X. X. , P. Yi , K. J. Chen , et al. 2018. “Knockdown of Arsenic Resistance Protein 2 Inhibits Human Glioblastoma Cell Proliferation Through the MAPK/ERK Pathway.” Oncology Reports 40: 3313–3322. 10.3892/or.2018.6777. PMC6196630 30542699

[mpp70359-bib-0024] Kurihara, Y. , and Y. Watanabe . 2004. “ *Arabidopsis* Micro‐RNA Biogenesis Through Dicer‐Like 1 Protein Functions.” Proceedings of the National Academy of Sciences of the United States of America 101: 12753–12758. 10.1073/pnas.0403115101. PMC515125 15314213

[mpp70359-bib-0025] Laubinger, S. , T. Sachsenberg , G. Zeller , et al. 2008. “Dual Roles of the Nuclear Cap‐Binding Complex and SERRATE in Pre‐mRNA Splicing and microRNA Processing in *Arabidopsis thaliana* .” Proceedings of the National Academy of Sciences of the United States of America 105: 8795–8800. 10.1073/pnas.0802493105. PMC2426964 18550839

[mpp70359-bib-0026] Liu, Y. , J. K. Liu , G. H. Li , et al. 2019. “A Novel *Botrytis cinerea*‐Specific Gene *BcHBF1* Enhances Virulence of the Grey Mould Fungus via Promoting Host Penetration and Invasive Hyphal Development.” Molecular Plant Pathology 20: 731–747. 10.1111/mpp.12788. PMC6637910 31008573

[mpp70359-bib-0027] Lobbes, D. , G. Rallapalli , D. D. Schmidt , C. Martin , and J. Clarke . 2006. “SERRATE: A New Player on the Plant microRNA Scene.” EMBO Reports 7: 1052–1058. 10.1038/sj.embor.7400806. PMC1618363 16977334

[mpp70359-bib-0028] Ma, L. , T. Liu , Z. Ke , et al. 2022. “ *Botrytis cinerea* Transcription Factor BcXyr1 Regulates (Hemi‐) Cellulase Production and Fungal Virulence.” mSystems 7: e0104222. 10.1128/msystems.01042-22. PMC9765177 36468854

[mpp70359-bib-0029] Marschall, R. , and P. Tudzynski . 2017. “The Protein Disulfide Isomerase of *Botrytis cinerea*: An ER Protein Involved in Protein Folding and Redox Homeostasis Influences NADPH Oxidase Signaling Processes.” Frontiers in Microbiology 8: 960. 10.3389/fmicb.2017.00960. PMC5447010 28611757

[mpp70359-bib-0030] Michlewski, G. , and J. F. Cáceres . 2019. “Post‐Transcriptional Control of miRNA Biogenesis.” RNA 25: 1–16. 10.1261/rna.068692.118. PMC6298569 30333195

[mpp70359-bib-0031] Porquier, A. , J. Moraga , G. Morgant , et al. 2019. “Botcinic Acid Biosynthesis in *Botrytis cinerea* Relies on a Subtelomeric Gene Cluster Surrounded by Relics of Transposons and Is Regulated by the Zn_2_Cys_6_ Transcription Factor BcBoa13.” Current Genetics 65: 965–980. 10.1007/s00294-019-00952-4. 30848345

[mpp70359-bib-0032] Porquier, A. , G. Morgant , J. Moraga , et al. 2016. “The Botrydial Biosynthetic Gene Cluster of *Botrytis cinerea* Displays a Bipartite Genomic Structure and Is Positively Regulated by the Putative Zn(II)_2_Cys_6_ Transcription Factor BcBot6.” Fungal Genetics and Biology 96: 33–46. 10.1016/j.fgb.2016.10.003. 27721016

[mpp70359-bib-0033] Prigge, M. J. , and D. R. Wagner . 2001. “The *Arabidopsis* Serrate Gene Encodes a Zinc‐Finger Protein Required for Normal Shoot Development.” Plant Cell 13: 1263–1279. 10.1105/tpc.13.6.1263. PMC135584 11402159

[mpp70359-bib-0034] Ren, G. , M. Xie , Y. Dou , S. Zhang , C. Zhang , and B. Yu . 2012. “Regulation of miRNA Abundance by RNA Binding Protein TOUGH in *Arabidopsis* .” Proceedings of the National Academy of Sciences of the United States of America 109: 12817–12821. 10.1073/pnas.1204915109. PMC3412041 22802657

[mpp70359-bib-0035] Rossman, T. G. , and Z. L. Wang . 1999. “Expression Cloning for Arsenite‐Resistance Resulted in Isolation of Tumor‐Suppressor cDNA: Possible Involvement of the Ubiquitin System in Arsenic Carcinogenesis.” Carcinogenesis 20: 311–316. 10.1093/carcin/20.2.311. 10069470

[mpp70359-bib-0036] Rouse, J. , and S. P. Jackson . 2002. “Interfaces Between the Detection, Signaling, and Repair of DNA Damage.” Science 297: 547–551. 10.1126/science.1074740. 12142523

[mpp70359-bib-0037] Singh, R. , C. Caseys , and D. J. Kliebenstein . 2024. “Genetic and Molecular Landscapes of the Generalist Phytopathogen *Botrytis cinerea* .” Molecular Plant Pathology 25: e13404. 10.1111/mpp.13404. PMC10788480 38037862

[mpp70359-bib-0038] Son, H. , M. Fu , Y. Lee , et al. 2016. “A Novel Transcription Factor Gene *FHS1* Is Involved in the DNA Damage Response in *Fusarium graminearum* .” Scientific Reports 6: 21572. 10.1038/srep21572. PMC4757864 26888604

[mpp70359-bib-0039] Su, L. Y. , T. Zhang , B. Yang , et al. 2024. “The *Botrytis cinerea* Effector BcXYG1 Suppresses Immunity in *Fragaria vesca* by Targeting FvBPL4 and FvACD11.” Horticulture Research 11: 251. 10.1093/hr/uhad251. PMC10831327 38304330

[mpp70359-bib-0040] Sun, H. Y. , H. C. Wang , Y. Chen , H. X. Li , and M. G. Zhou . 2010. “Multiple Resistance of *Botrytis cinerea* From Vegetable Crops to Carbendazim, Diethofencarb, Procymidone, and Pyrimethanil in China.” Plant Disease 94: 551–556. 10.1094/PDIS-94-5-0551. 30754466

[mpp70359-bib-0041] Sun, J. , C. H. Sun , H. W. Chang , et al. 2021. “Cyclophilin BcCyp2 Regulates Infection‐Related Development to Facilitate Virulence of the Gray Mold Fungus *Botrytis cinerea* .” International Journal of Molecular Sciences 22: 1694. 10.3390/ijms22041694. PMC7914984 33567582

[mpp70359-bib-0042] Tang, M. Y. , K. X. Wang , P. P. Zhang , et al. 2024. “Establishment of a Mutant Library for Infection Cushion Development and Identification of a Key Regulatory Gene in *Botrytis cinerea* .” Journal of Fungi 11: 16. 10.3390/jof11010016. PMC11766268 39852435

[mpp70359-bib-0043] Wang, H. Y. , Z. Y. Gao , X. S. Chen , et al. 2023. “ *BcWRKY22* Activates *BcCAT2* to Enhance Catalase (CAT) Activity and Reduce Hydrogen Peroxide (H_2_O_2_) Accumulation, Promoting Thermotolerance in Non‐Heading Chinese Cabbage ( *Brassica campestris* ssp. *chinensis*).” Antioxidants 12: 1710. 10.3390/antiox12091710. PMC10525746 37760013

[mpp70359-bib-0044] Wang, M. , A. Weiberg , E. Dellota Jr. , D. Yamane , and H. L. Jin . 2017. “ *Botrytis* Small RNA Bc‐siR37 Suppresses Plant Defense Genes by Cross‐Kingdom RNAi.” RNA Biology 14: 421–428. 10.1080/15476286.2017.1291112. PMC5411126 28267415

[mpp70359-bib-0045] Wang, M. , A. Weiberg , F. M. Lin , B. Thomma , H. D. Huang , and H. L. Jin . 2016. “Bidirectional Cross‐Kingdom RNAi and Fungal Uptake of External RNAs Confer Plant Protection.” Nature Plants 2: 16151. 10.1038/nplants.2016.151. PMC5040644 27643635

[mpp70359-bib-0046] Weiberg, A. , M. Wang , F. M. Lin , et al. 2013. “Fungal Small RNAs Suppress Plant Immunity by Hijacking Host RNA Interference Pathways.” Science 342: 118–123. 10.1126/science.1239705. PMC4096153 24092744

[mpp70359-bib-0047] Wu, F. , L. Yu , W. Cao , Y. Mao , Z. Liu , and Y. He . 2007. “The N‐Terminal Double‐Stranded RNA Binding Domains of *Arabidopsis* HYPONASTIC LEAVES1 Are Sufficient for Pre‐microRNA Processing.” Plant Cell 19: 914–925. 10.1105/tpc.106.048637. PMC1867364 17337628

[mpp70359-bib-0048] Yang, L. , Z. Liu , F. Lu , A. Dong , and H. Huang . 2006. “SERRATE Is a Novel Nuclear Regulator in Primary microRNA Processing in *Arabidopsis* .” Plant Journal 47: 841–850. 10.1111/j.1365-313X.2006.02835.x. 16889646

[mpp70359-bib-0049] Yu, B. , L. Bi , B. Zheng , et al. 2008. “The FHA Domain Proteins DAWDLE in *Arabidopsis* and SNIP1 in Humans Act in Small RNA Biogenesis.” Proceedings of the National Academy of Sciences of the United States of America 105: 10073–10078. 10.1073/pnas.0804218105. PMC2481372 18632581

[mpp70359-bib-0050] Yuan, L. , X. X. Jiang , Q. H. Gong , and N. Gao . 2023. “Arsenic Resistance Protein 2 and microRNA Biogenesis: Biological Implications in Cancer Development.” Pharmacology & Therapeutics 244: 108386. 10.1016/j.pharmthera.2023.108386. 36933704

[mpp70359-bib-0051] Zhang, M. Z. , C. H. Sun , Y. Liu , et al. 2020. “Transcriptome Analysis and Functional Validation Reveal a Novel Gene, *BcCGF1*, That Enhances Fungal Virulence by Promoting Infection‐Related Development and Host Penetration.” Molecular Plant Pathology 21: 834–853. 10.1111/mpp.12934. PMC7214349 32301267

[mpp70359-bib-0052] Zhang, Y. S. , X. T. Chen , G. H. Li , Q. M. Qin , M. Z. Zhang , and J. C. Qin . 2025. “CBC Complex Regulates Hyphal Growth, Sclerotial Quantity, and Pathogenicity in the Necrotrophic Fungus *Botrytis cinerea* .” Journal of Fungi 11: 429. 10.3390/jof11060429. PMC12194085 40558941

[mpp70359-bib-0053] Zhang, Y. S. , C. G. Jia , Y. Liu , et al. 2024. “The Fungal Transcription Factor BcTbs1 From *Botrytis cinerea* Promotes Pathogenicity via Host Cellulose Degradation.” Journal of Agricultural and Food Chemistry 72: 20816–20830. 10.1021/acs.jafc.4c03744. 39261294

[mpp70359-bib-0054] Zhang, Y. S. , L. M. Wang , S. Liang , et al. 2020. “FpDep1, a Component of Rpd3L Histone Deacetylase Complex, Is Important for Vegetative Development, ROS Accumulation, and Pathogenesis in *Fusarium pseudograminearum* .” Fungal Genetics and Biology 135: 103299. 10.1016/j.fgb.2019.103299. 31706014

[mpp70359-bib-0055] Zhou, B. B. S. , and S. J. Elledge . 2000. “The DNA Damage Response: Putting Checkpoints in Perspective.” Nature 408: 433–439. 10.1038/35044005. 11100718

[mpp70359-bib-0056] Zhu, C. X. , Y. Tang , D. D. Ren , et al. 2023. “Propionate Poses Antivirulence Activity Against *Botrytis cinerea* via Regulating Its Metabolism, Infection Cushion Development and Overall Pathogenic Factors.” Food Chemistry 410: 135443. 10.1016/j.foodchem.2023.135443. 36680882

