# Supplementary material for: Arsenic Resistance Protein BcArs2 Modulates Endogenous ROS Homeostasis in Infection Cushions to Enhance the Virulence of Botrytis cinerea Toward Host Plants
Source: Mol Plant Pathol. 2026 Oct 13;27(10):e70359. doi: 10.1111/mpp.70359 (PMC13659265; doi:10.1111/mpp.70359)
Supplement: Supplementary file 1 [file MPP-27-e70359-s002.docx]

**Figure S8. Exogenous hydrogen peroxide promotes infection‑cushion maturation in the mutant.**

(A) Observation of infection cushions formed by tested strains upon supplementation with 7.5 mM H_2_O_2_. Scale bar = 40 µm.

(B) Quantification of the relative number of infection cushions in the indicated strains.

(C) Quantification of the relative size of infection cushions in the indicated strains.

Data represent means ± SD from three independent experiments, in which triplicate slides were examined for each strain per experiment. Asterisks indicate significant differences based on Student’s *t*-test. ** and *** indicate *p* < 0.01 and *p* < 0.001, respectively.
