# Supplementary material for: Arsenic Resistance Protein BcArs2 Modulates Endogenous ROS Homeostasis in Infection Cushions to Enhance the Virulence of Botrytis cinerea Toward Host Plants
Source: Mol Plant Pathol. 2026 Oct 13;27(10):e70359. doi: 10.1111/mpp.70359 (PMC13659265; doi:10.1111/mpp.70359)
Supplement: Supplementary file 2 [file MPP-27-e70359-s004.docx]

**Figure S2. Construction and identification of *BcARS2* knockout, complementation, and GFP fusion strains.**

(A-B) Schematic diagrams of the BcArs2::GFP fusion construct and the GFP control construct. The BcArs2::GFP fusion construct driven by the *Olic* promoter was transformed into the WT strain (A); the GFP control construct driven by the *Olic* promoter was transformed into the WT strain (B).

(C-D) Strategy for the construction of *BcARS2* gene disruption (Δ*Bcars2*) mutant and complementation (Δ*Bcars2*-C) strains.

(E) The PCR detection results for the single-spore isolation*.* The images are presented in order: PCR after primary hygromycin screening; PCR after first single-spore isolation; PCR after second single-spore isolation. Note: M represents DL5000 DNA Marker (Vazyme, MD102-01).

(F) Southern blot analysis of *Age*I-digested genomic DNA from WT and Δ*Bcars2* strains. The *HPH* probe detected the expected 3611 bp bands in Δ*Bcars2*-13 and Δ*Bcars2*-27, whereas the *BcARS2* probe detected the expected 1662 bp band in the WT strain. Restriction enzyme sites and probe positions are indicated in panel (C). Note: M1 indicates a 10 kb DNA Ladder (Sangon Biotech, B600032), and M2 indicates a DL2000 DNA marker (Sangon Biotech, B600335).

(G) PCR detection of *BcARS2* in the tested strains. A 1465 bp fragment of *BcARS2* was amplified with the primer pair Ars2‑NF/Ars2‑NR from the genomes of the WT and Δ*Bcars2‑*C complemented strains; this fragment was absent in the Δ*Bcars2* mutant strains. Note: M represents DL2000 Plus DNA Marker (Vazyme, MD101-01).

(H) Semi-quantitative PCR detection of *BcARS2* expression in WT, Δ*Bcars2*-13, Δ*Bcars2*-27, and Δ*Bcars2*-C strains. *BcACT* was amplified as an internal reference, yielding a 160 bp band. A 110 bp fragment of *BcARS2* was detected in the WT and Δ*Bcars2*-C strains. Note: M indicates DL2000 DNA Marker (Sangon Biotech, B600335).
