# Supplementary material for: Arsenic Resistance Protein BcArs2 Modulates Endogenous ROS Homeostasis in Infection Cushions to Enhance the Virulence of Botrytis cinerea Toward Host Plants
Source: Mol Plant Pathol. 2026 Oct 13;27(10):e70359. doi: 10.1111/mpp.70359 (PMC13659265; doi:10.1111/mpp.70359)
Supplement: Supplementary file 3 [file MPP-27-e70359-s006.docx]

**Figure S4. Conidial germination of the indicated strains.**

(A) Conidial germination of the WT, Δ*Bcars2*-13, Δ*Bcars2*-27, and Δ*Bcars2*-C strains at the indicated time points. Scale bar = 10 μm.
(B) Quantification of relative germination rates at the indicated hours post-inoculation (hpi).
(C) Quantification of relative germ tube (GT) lengths at the indicated hpi. Data represent means ± SD from three independent experiments, in which triplicate slides were examined for each strain per experiment. Asterisks indicate significant differences based on Student’s *t*-test. ** indicates *p* < 0.01.
