# Supplementary material for: Arsenic Resistance Protein BcArs2 Modulates Endogenous ROS Homeostasis in Infection Cushions to Enhance the Virulence of Botrytis cinerea Toward Host Plants
Source: Mol Plant Pathol. 2026 Oct 13;27(10):e70359. doi: 10.1111/mpp.70359 (PMC13659265; doi:10.1111/mpp.70359)
Supplement: Supplementary file 4 [file MPP-27-e70359-s008.docx]

**Figure S3 Cluster analysis of the expression of miRNA processing-related genes upon *BcARS2* knockout.**
