# Supplementary material for: Arsenic Resistance Protein BcArs2 Modulates Endogenous ROS Homeostasis in Infection Cushions to Enhance the Virulence of Botrytis cinerea Toward Host Plants
Source: Mol Plant Pathol. 2026 Oct 13;27(10):e70359. doi: 10.1111/mpp.70359 (PMC13659265; doi:10.1111/mpp.70359)
Supplement: Supplementary file 5 [file MPP-27-e70359-s010.docx]

**Figure S5. Pathogenicity assay on tobacco leaves.**

(A-B) Disease symptoms on tobacco leaves caused by the indicated strains during the infection time course via spore inoculation (A) or mycelial plug inoculation (B).

(C-D) Quantification of lesion sizes caused by the indicated strains as shown in (A) and (B).

Data represent means ± SD from three independent experiments, in which triplicate leaves were examined for each strain per experiment. Asterisks indicate significant differences based on Student’s *t*-test. *** indicates *p* < 0.001. ND indicates no data detected.
