# Supplementary material for: Arsenic Resistance Protein BcArs2 Modulates Endogenous ROS Homeostasis in Infection Cushions to Enhance the Virulence of Botrytis cinerea Toward Host Plants
Source: Mol Plant Pathol. 2026 Oct 13;27(10):e70359. doi: 10.1111/mpp.70359 (PMC13659265; doi:10.1111/mpp.70359)
Supplement: Supplementary file 6 [file MPP-27-e70359-s011.docx]

Table S6 18 genes associated with infection cushion development

| GeneID | Locus tag | Mutant VS WT | IC VS Myc | Infection cushions phenotype in the mutant |
| --- | --- | --- | --- | --- |
| *BcSEP4* (Feng et al., 2017) | *BCIN_04g05920* | no significant | no significant | Failure to form ICs |
| *BcNOP53* (Cao et al., 2018) | *BCIN_12g03770* | up-regulate | down-regulate | Failure to form ICs |
| *BcCGF1* (Zhang et al., 2020a) | *BCIN_16g01820* | down-regulate | up-regulate | Reduced ICs number |
| *BcJAR1* (Hou et al., 2020) | *BCIN_02g00190* | no significant | no significant | Failure to form ICs |
| *BcMSB2* (Leroch et al., 2015) | *BCIN_12g01370* | down-regulate | no significant | Failure to form ICs |
| *BcIQG1* (Marschall et al., 2016) | *BCIN_07g00180* | no significant | no significant | Failure to form ICs |
| *BcPDI1* (Marschall et al., 2017) | *BCIN_06g05730* | down-regulate | up-regulate | Failure to form ICs |
| *BcHBF1* (Liu et al., 2019) | *BCIN_06g00240* | no significant | down-regulate | Reduced number and size |
| *BcRPD3* (Zhang et al., 2020b) | *BCIN_05g02590* | no significant | no significant | Reduced number at the early stage |
| *BcEXO70* (Tang et al., 2024) | *BCIN_03g07900* | no significant | no significant | Failure to form ICs |
| *BcTBS1* (Zhang et al., 2024) | *BCIN_13g03910* | no significant | no significant | Reduced total IC biomass |
| *BcSER2* (Liu et al., 2020) | *BCIN_08g02990* | down-regulate | no significant | Failure to form ICs |
| *BcGBL1* (Tang et al., 2023) | *BCIN_14g03010* | no significant | no significant | Failure to form ICs |
| *BcCCC2* (Saitoh et al., 2010) | *BCIN_05g00240* | down-regulate | no significant | Abnormal morphology and reduced number |
| *BcWCL2* (Ren et al., 2024) | *BCIN_05g05530* | no significant | no significant | Reduced number |
| *BcGB1* (Tang et al., 2021) | *BCIN_08g01420* | no significant | no significant | Reduced number |
| *Bac* (Chen et al., 2020) | *BCIN_15g02590* | no significant | no significant | Reduced number |
| *BcSpd1* (Chen et al., 2022) | *BCIN_06g05230* | no significant | no significant | Reduced number |

References

Cao, S.N., Yuan, Y., Qin, Y.H., et al. 2018. “The pre-rRNA processing factor Nop53 regulates fungal development and pathogenesis via mediating production of reactive oxygen species.” *Environmental Microbiology* **20**: 1531-1549. http://dx.doi.org/10.1111/1462-2920.14082.

Chen, H.C., He, S.N., Zhang, S.H., A, R., Li, W.L., and Liu, S.A. 2022. “The necrotroph *Botrytis cinerea* BcSpd1 plays a key role in modulating both fungal pathogenic factors and plant disease development.” *Frontiers in plant science* **13**: 820767. http://dx.doi.org/10.3389/fpls.2022.820767.

Chen, X., Zhang, X.H., Zhu, P.K., et al. 2020. “A single nucleotide mutation in adenylate cyclase affects vegetative growth, sclerotial formation and virulence of *Botrytis cinerea*.” *International Journal of Molecular Sciences* **21**: 2912. http://dx.doi.org/10.3390/ijms21082912.

Feng, H.Q., Li, G.H., Du, S.W., et al. 2017. “The septin protein Sep4 facilitates host infection by plant fungal pathogens via mediating initiation of infection structure formation.” *Environmental Microbiology* **19**: 1730-1749. http://dx.doi.org/10.1111/1462-2920.13613.

Hou, J., Feng, H.Q., Chang, H.W., et al. 2020. “The H3K4 demethylase Jar1 orchestrates ROS production and expression of pathogenesis-related genes to facilitate *Botrytis cinerea* virulence.” *New Phytologist* **225**: 930-947. http://dx.doi.org/10.1111/nph.16200.

Leroch, M., Mueller, N., Hinsenkamp, I., and Hahn, M. 2015. “The signalling mucin Msb2 regulates surface sensing and host penetration via BMP1 MAP kinase signalling in *Botrytis cinerea*.” *Molecular Plant Pathology* **16**: 787-798. http://dx.doi.org/10.1111/mpp.12234.

Liu, X.Q., Xie, J.T., Fu, Y.P., Jiang, D.H., Chen, T., and Cheng, J.S. 2020. “The Subtilisin-Like protease BcSer2 affects the sclerotial formation, conidiation and virulence of *Botrytis cinerea*.” *International Journal of Molecular Sciences* **21**: 603. http://dx.doi.org/10.3390/ijms21020603.

Liu, Y., Liu, J.K., Li, G.H., et al. 2019. “A novel *Botrytis cinerea*-specific gene *BcHBF1* enhances virulence of the grey mould fungus via promoting host penetration and invasive hyphal development.” *Molecular Plant Pathology* **20**: 731-747. http://dx.doi.org/10.1111/mpp.12788.

Marschall, R., and Tudzynski, P. 2016. “BcIqg1, a fungal IQGAP homolog, interacts with NADPH oxidase, MAP kinase and calcium signaling proteins and regulates virulence and development in *Botrytis cinerea*.” *Molecular Microbiology* **101**: 281-298. http://dx.doi.org/10.1111/mmi.13391.

Marschall, R., and Tudzynski, P. 2017. “The protein disulfide isomerase of *Botrytis cinerea*: An ER protein involved in protein folding and redox homeostasis influences NADPH oxidase signaling processes.” *Frontiers in microbiology* **8**: 960. http://dx.doi.org/10.3389/fmicb.2017.00960.

Ren, W.H., Qian, C., Ren, D.D., et al. 2024. “The GATA transcription factor *BcWCL2* regulates citric acid secretion to maintain redox homeostasis and full virulence in *Botrytis cinerea*.” *mBio* **15**: e0013324. http://dx.doi.org/10.1128/mbio.00133-24.

Saitoh, Y., Izumitsu, K., Morita, A., and Tanaka, C. 2010. “A copper-transporting ATPase *BcCCC2* is necessary for pathogenicity of *Botrytis cinerea*.” *Molecular Genetics and Genomics* **284**: 33-43. http://dx.doi.org/10.1007/s00438-010-0545-4.

Tang, J.J., Sui, Z., Li, R.H., et al. 2023. “The Gβ-like protein Bcgbl1 regulates development and pathogenicity of the gray mold *Botrytis cinerea* via modulating two MAP kinase signaling pathways.” *PLoS Pathogens* **19**: e1011839. http://dx.doi.org/10.1371/journal.ppat.1011839.

Tang, J.J., Wu, M.D., Zhang, J., Li, G.Q., and Yang, L. 2021. “*Botrytis cinerea* G protein β subunit Bcgb1 controls growth, development and virulence by regulating cAMP signaling and MAPK signaling.” *Journal of Fungi* **7**: 431. http://dx.doi.org/10.3390/jof7060431.

Tang, M.Y., Wang, K.X., Zhang, P., et al. 2024. “Establishment of a mutant library for infection cushion development and identification of a key regulatory gene in *Botrytis cinerea*.” *Journal of Fungi* **11**: 16. http://dx.doi.org/10.3390/jof11010016.

Zhang, M.Z., Sun, C.H., Liu, Y., et al. 2020a. “Transcriptome analysis and functional validation reveal a novel gene, *BcCGF1*, that enhances fungal virulence by promoting infection-related development and host penetration.” *Molecular Plant Pathology* **21**: 834-853. http://dx.doi.org/10.1111/mpp.12934.

Zhang, N., Yang, Z.Z., Zhang, Z.H., and Liang, W.X. 2020b. “BcRPD3-mediated histone deacetylation is involved in growth and pathogenicity of *Botrytis cinerea*.” *Frontiers in microbiology* **11**: 1832. http://dx.doi.org/10.3389/fmicb.2020.01832.

Zhang, Y.S., Liu, Y., Jia, C.G., et al. 2024. “The Fungal transcription factor BcTbs1 from *Botrytis cinerea* promotes pathogenicity via host cellulose degradation.” *Journal of Agricultural and Food Chemistry* **72**: 20816-20830. http://dx.doi.org/10.1021/acs.jafc.4c09198.
