# Supplementary material for: Arsenic Resistance Protein BcArs2 Modulates Endogenous ROS Homeostasis in Infection Cushions to Enhance the Virulence of Botrytis cinerea Toward Host Plants
Source: Mol Plant Pathol. 2026 Oct 13;27(10):e70359. doi: 10.1111/mpp.70359 (PMC13659265; doi:10.1111/mpp.70359)
Supplement: Supplementary file 7 [file MPP-27-e70359-s012.docx]

**Figure S1. Bioinformatics analysis of Ars2*.***

(A) Phylogenetic analysis of Ars2 homologs from diverse organisms. The included species and their corresponding protein accession numbers are as follows: *Drosophila melanogaster* (NP_724455.2), *Homo sapiens* (AAI09118.1), *Caenorhabditis elegans* (CCD68572.1), *Schizosaccharomyces pombe* (NP_595488.1), *Fusarium graminearum* (XP_011316870.1), *Botrytis cinerea* (XP_001558938.1), *Sclerotinia sclerotiorum* (XP_001597623.1), and *Arabidopsis thaliana* (NP_565635.1).

(B) Conserved domain analysis of Ars2 homologs from related organisms. Two conserved domains were identified: DUF3456 (an uncharacterized functional domain) and ARS2 domain (a domain associated with arsenite resistance).

(C) Predicted three-dimensional structures of FgArs2 and BcArs2 generated by SWISS-MODEL.
