# Supplementary material for: Arsenic Resistance Protein BcArs2 Modulates Endogenous ROS Homeostasis in Infection Cushions to Enhance the Virulence of Botrytis cinerea Toward Host Plants
Source: Mol Plant Pathol. 2026 Oct 13;27(10):e70359. doi: 10.1111/mpp.70359 (PMC13659265; doi:10.1111/mpp.70359)
Supplement: Supplementary file 8 [file MPP-27-e70359-s013.docx]

**Figure S6. Pathogenicity analysis of the indicated strains on wounded and unwounded leaf tissues.**

(A-B) Disease symptoms on wounded and unwounded green bean leaves inoculated with conidial suspensions (A) or mycelial plugs (B) of the WT and Δ*Bcars2*-13.

(C-D) Disease symptoms on wounded and unwounded tobacco leaves inoculated with conidial suspensions (C) or mycelial plugs (D) of the WT and Δ*Bcars2-*13.

(E-H) Quantification of lesion sizes caused by the indicated strains, corresponding to (A-D), respectively.

Data represent means ± SD from three independent experiments, in which triplicate leaves were examined for each strain per experiment. Asterisks indicate significant differences based on Student’s *t*-test. * and *** indicate significance at *p* < 0.05 and *p* < 0.001, respectively. ND indicates no data detected. Red arrows indicate lesions.
