# Supplementary material for: Arsenic Resistance Protein BcArs2 Modulates Endogenous ROS Homeostasis in Infection Cushions to Enhance the Virulence of Botrytis cinerea Toward Host Plants
Source: Mol Plant Pathol. 2026 Oct 13;27(10):e70359. doi: 10.1111/mpp.70359 (PMC13659265; doi:10.1111/mpp.70359)
Supplement: Supplementary file 9 [file MPP-27-e70359-s014.docx]

**Figure S7. Analysis of differentially expressed genes (DEGs).**

(A) Volcano plots of DEGs between the WT and Δ*Bcars2* strains. Each dot represents a gene.

(B) KEGG pathway classification of DEGs in the WT versus Δ*Bcars2* comparison.

(C) Functional enrichment analysis of DEGs upregulated in ICs relative to mycelia but downregulated in Δ*Bcars2* relative to the WT.

(D) Functional enrichment analysis of DEGs downregulated in ICs relative to mycelia but upregulated in Δ*Bcars2* relative to the WT.
